# Type-I collagen produced by distinct fibroblast lineages reveals specific function during embryogenesis and Osteogenesis Imperfecta

**DOI:** 10.1038/s41467-021-27563-3

**Published:** 2021-12-10

**Authors:** Yang Chen, Sujuan Yang, Sara Lovisa, Catherine G. Ambrose, Kathleen M. McAndrews, Hikaru Sugimoto, Raghu Kalluri

**Affiliations:** 1 Department of Cancer Biology, University of Texas MD Anderson Cancer Center, Houston, TX 77054 USA; 2 Department of Orthopaedic Surgery, University of Texas Health Science Center at Houston, Houston, TX USA; 3 Department of Bioengineering, Rice University, Houston, TX USA; 4 Department of Molecular and Cellular Biology, Baylor College of Medicine, Houston, TX USA

**Keywords:** Bone development, Cell lineage, Extracellular matrix

## Abstract

Type I collagen (Col1) is the most abundant protein in mammals. Col1 contributes to 90% of the total organic component of bone matrix. However, the precise cellular origin and functional contribution of Col1 in embryogenesis and bone formation remain unknown. Single-cell RNA-sequencing analysis identifies Fap^+^ cells and Fsp1^+^ cells as the major contributors of Col1 in the bone. We generate transgenic mouse models to genetically delete Col1 in various cell lineages. Complete, whole-body Col1 deletion leads to failed gastrulation and early embryonic lethality. Specific Col1 deletion in Fap^+^ cells causes severe skeletal defects, with hemorrhage, edema, and prenatal lethality. Specific Col1 deletion in Fsp1^+^ cells results in Osteogenesis Imperfecta-like phenotypes in adult mice, with spontaneous fractures and compromised bone healing. This study demonstrates specific contributions of mesenchymal cell lineages to Col1 production in organogenesis, skeletal development, and bone formation/repair, with potential insights into cell-based therapy for patients with Osteogenesis Imperfecta.

## Abstract (web summary)

Collagen is the most abundant protein in the human body. Here, the authors show that different classes of fibroblasts produce collagen of unique functions with different impacts on embryo development and bone formation.

## Introduction

Type I collagen (Col1), a fibrillar collagen, is the most abundant protein in mammals. The basic functional unit of Col1 is a heterotrimer consisting of two α1 chains and one α2 chain that assemble together to form a triple helical structure. Each α-chain polypeptide is synthesized in the cytosol and combines with two other α-chains to generate a triple-helical type I procollagen (in the format of α1α2α1) with N-terminal and C-terminal propeptides. Subsequently, the procollagen molecule is secreted into the extracellular space where the N-terminal and C-terminal propeptides are cleaved by propeptidases, generating the basic functional heterotrimeric unit of Col1. The Col1 triple helical rod-like molecules interact with each other to form fibrils and undergo further crosslinking to form large bundles of fibers.

During embryogenesis, many organs express Col1 to likely facilitate cellular migration, differentiation and structural compartmentalization^1^. Insertional mutation of *Col1a1* gene resulted in complete absence of Col1 and led to embryonic lethality^2^. Genetic mutations in *Col1a1* or *Col1a2* genes (or other genes affecting Col1 biosynthesis) in humans lead to multiple subtypes of Osteogenesis Imperfecta (OI) syndrome, a bone disease referred to as the ‘brittle bone disease’^3–5^. Previous studies established and investigated various Osteogenesis Imperfecta mouse models using different genetic targeting/manipulating approaches with some success^6–19^. Particularly, recent studies demonstrated that *Col1a1* heterozygous mutation/deletion in transgenic mouse models lead to Osteogenesis Imperfecta phenotypes^14,19^.

Col1 contributes around 90% of the total organic component of bone matrix. The development, formation, and homeostasis of bone tissue involve mesenchymal cell lineages in the synthesis, deposition, and remodeling of Col1^4,20^. These mesenchymal cell lineages include osteoblasts and fibroblasts, which can be differentiated from mesenchymal stem/stromal cells (MSCs)^3,4,21–25^. However, the precise contributions of Col1 produced by these mesenchymal cell lineages and fibroblasts in the development and function of bones remain unknown. Recent studies, using single-cell RNA sequencing analysis (sc-RNA-seq) and other techniques, highlighted the distinct features and potential functions of various osteogenic cell subpopulations in the bone and bone marrow, shedding some light on the potential mechanisms driving bone homeostasis and repair^26–28^. In this present study, we analyzed sc-RNA-seq dataset on bone and bone marrow stroma of healthy mice^27^, and identified the expression of *Col1a1* gene dominantly in Fap^+^ (fibroblast activation protein; encoded by *Fap*) mesenchymal cells, as well as S100a4^+^ (also known as fibroblast-specific protein 1, Fsp1; encoded by *S100a4*) mesenchymal cells. Next, we generated novel genetically engineered mouse models (GEMMs) allowing the conditional deletion of Col1 specifically in Fap^+^ or Fsp1^+^ cells, as well as in other cell lineages. The *Col1a1*^*loxP/loxP*^ mouse strain was crossed with multiple Cre recombinase mouse strains including *CMV-Cre*, *Fap-Cre*, *Fsp1 (S100a4)-Cre*, *αSMA (Acta2)-Cre,* and *Cdh5-Cre* strains. These mice revealed distinct phenotypes due to Cre recombinase-driven Col1 deletion in targeted cell lineages. Taken together, this study provides comprehensive investigation of the functional roles of Col1 produced by various cell lineages in organogenesis, osteogenesis, and bone-related diseases.

## Results

### Analysis of single-cell RNA-sequencing of mouse bone and bone marrow identifies type I collagen (Col1)-producing fibroblast/mesenchymal lineages

**Fig. 1:**
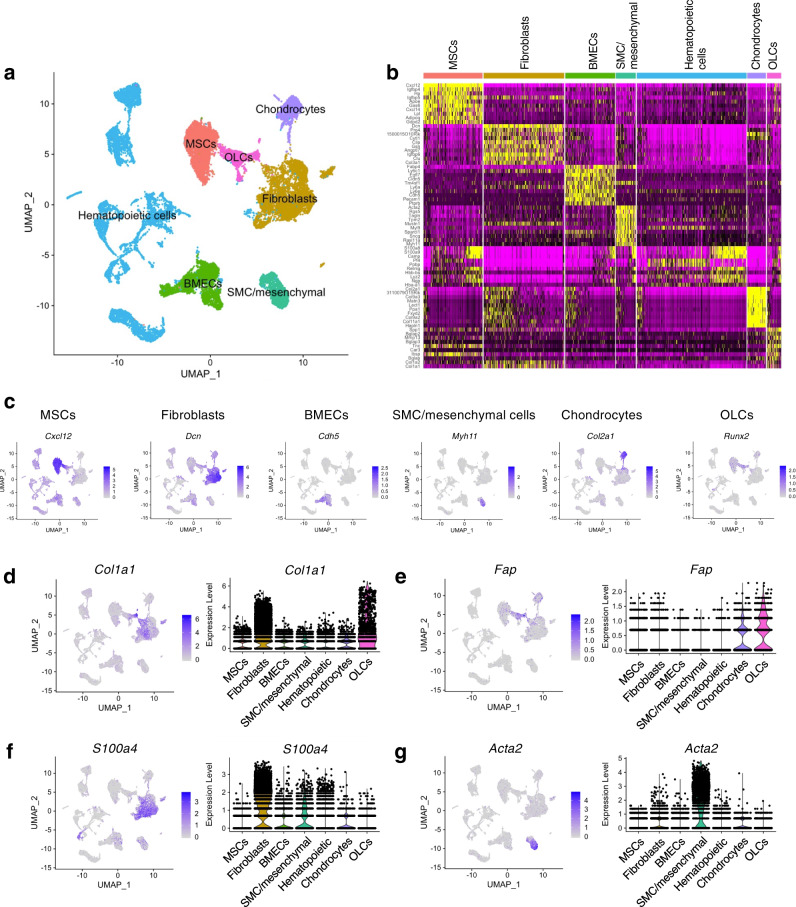
Single-cell RNA-sequencing analysis identifies Col1-producing cells/lineages in bone and bone marrow fractions. **a**–**c** Single-cell RNA-sequencing (sc-RNA-seq) analysis of cell mixture of mouse bone and bone marrow fractions (*n* = 6 mice) from a recently published dataset (GSE128423). Functional clusters of cells were defined with group definition listed, as implemented in the Seurat R package (**a**). MSCs mesenchymal stem/stromal cells, OLCs osteolineage cells derived from MSCs, BMECs bone marrow endothelial cells, SMC/mesenchymal smooth-muscle cell/mesenchymal lineage. **b** Heat map showing the scaled expression values of discriminating signature genes for the functional clusters of cells defined above. (**c**) Expression profile of representative signature gene for each of the non-hematopoietic cell clusters. Continued in Supplementary Fig. 1 and 2. **d**–**g** Expression profile of type I collagen α1 chain (*Col1a1*; **d**), fibroblast activation protein (*Fap*; **e**), fibroblast-specific protein 1 (*S100a4*; **f**), and α-smooth muscle actin (*Acta2*; **g**) among defined cell clusters shown in UMAP plot and violin plot.

Bone marrow is one of the sources of mesenchymal lineages, pluripotent cells that differentiate into a variety of cell types such as osteoblasts, chondrocytes, and fibroblasts^3,4,21–25^. To identify the cell populations involved in type I collagen (Col1) production in osteogenesis, we further analyzed the single-cell RNA-sequencing data^27^ of combined cells from mouse bone (including the periosteum) and the bone marrow (Fig. 1a, Supplementary Fig. S1 and S2). Consistent with previous report^27^, six distinct non-hematopoietic cell populations were identified in the bone and the bone marrow (Fig. 1a–c, Supplementary Fig. S1). These are MSCs (expressing *Lepr* and *Cxcl12*, also called Cxcl12-abundant-reticular cells)^28–30^, MSC-descendent osteolineage cells (OLCs; expressing *Bglap* and *Runx2*)^31,32^, chondrocytes (expressing *Acan* and *Col2a1*)^33,34^, fibroblasts (expressing *S100a4* and *Dcn*)^35^, bone marrow endothelial cells (BMECs; expressing *Cdh5*, encoding VE-cadherin)^36^, and smooth-muscle cell/mesenchymal lineage (SMC/mesenchymal) (expressing *Acta2* and *Myh11*)^37^.

Next, we determined that *Col1a1* is highly expressed by MSC-descendent OLCs and fibroblasts (Fig. 1d). We simultaneously examined the expression of several genes encoding common fibroblast/mesenchymal markers, such as *Fap* (encoding fibroblast activation protein, Fap), *S100a4* (encoding Fibroblast-specific protein 1, Fsp1), and *Acta2* (encoding α-smooth muscle actin, αSMA), among the cell populations of bone and bone marrow. *Fap* expression was observed in cell clusters defined as MSC-descendent OLCs (Fig. 1e), and also observed in chondrocytes, fibroblasts, and MSCs. *Fap* expression in OLCs was observed in the matrix-producing mature osteoblast subpopulation expressing *Bglap* (encoding osteocalcin) (Supplementary Fig. 1b), and also the osteoprogenitor cell subpopulation expressing *Sp7* (Osterix) and *Mmp13* (Supplementary Fig. 1c)^27,28^. *S100a4* (Fsp1) expression was dominantly detected in all fibroblast clusters^27,35^, but also detected in SMC/mesenchymal cells, chondrocytes, and endothelial cells (Fig. 1f). *Acta2* (αSMA) was predominantly expressed in the SMC/mesenchymal cells, and was expressed in other cell types such as fibroblasts, chondrocytes, or osteolineage cells (Fig. 1g). It is important to note that *Col1a1*-expressing cell populations were largely constituted of Fap-expressing cells and Fsp1-expressing cells. *Col1a1* expression in OLCs was found predominantly in differentiated osteoblasts expressing *Bglap* (Fig. 1d, Supplementary Fig. 1b), but sparsely in osteoprogenitor cells expressing *Sp7* and *Mmp13* (Supplementary Fig. 1c), which is consistent with recent observations^26,27^. Taken together, these results suggest that Fap-expressing cells and Fsp1-expressing cells are likely associated with Col1 synthesis in the bones, prompting us to further investigate the functional role of Col1 produced by these cell lineages.

### Systemic deletion of *Col1a1* employing CMV-Cre results in failed gastrulation and early embryonic lethality

**Fig. 2:**
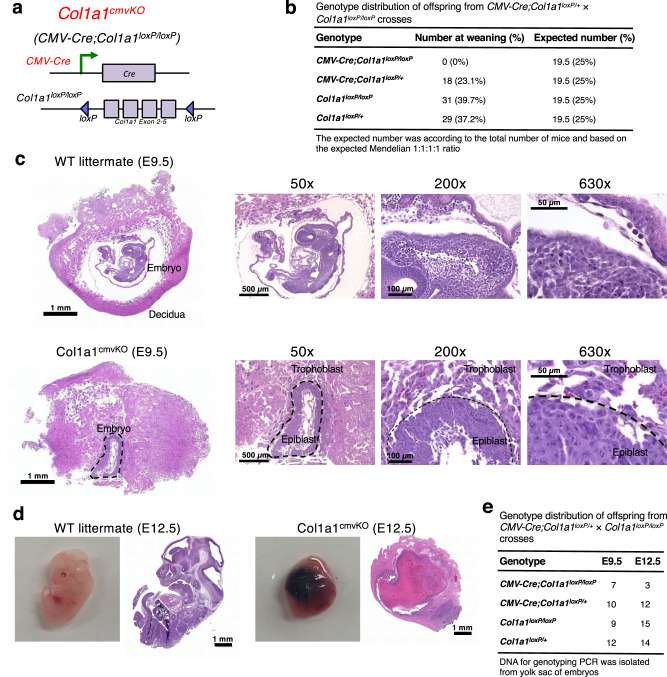
Systemic deletion of Col1 by CMV-Cre transgene results in early embryonic lethality. **a** Genetic strategy to achieve systemic (whole-body) knockout of type I collagen α1 chain (*Col1a1*) by crossing the *Col1a1*^*loxP/loxP*^ mice with generic *CMV-Cre* mice. **b** Genotype distribution in live offspring examined at the time of weaning from the crosses between *CMV-Cre;Col1a1*^*loxP/+*^ and *Col1a1*^*loxP/loxP*^ mice. **c**, **d** Hematoxylin and eosin (H&E) staining of the embryos of *CMV-Cre-negative;Col1a1*^*loxP/loxP*^ (WT; wild-type) and *CMV-Cre;Col1a1*^*loxP/loxP*^ (Col1a1^cmvKO^) at embryonic day E9.5 (**c**) and E12.5 (**d**). Col1a1^cmvKO^ embryo of E9.5 was indicated by dashed line, with labeled epiblast and trophoblast areas. Representative images were shown for WT embryos (*n* = 6) and Col1a1^cmvKO^ embryos (*n* = 6) of E9.5 (**c**); and WT embryos (*n* = 6) and Col1a1^cmvKO^ embryos (*n* = 3, limited number of obtained embryos due to lethality) of E12.5 (**d**). **e** Genotype distribution in live offspring examined at E9.5 and E12.5 from the crosses between *CMV-Cre;Col1a1*^*loxP/+*^ and *Col1a1*^*loxP/loxP*^ mice. Scale bars of whole-mount sections, 1 mm; Scale bars at 50× magnification, 500 μm; Scale bars at 200× magnification, 100 μm; Scale bars at 630× magnification, 50 μm.

We generated *Col1a1*^*loxP/loxP*^ mice allowing for conditional deletion of floxed *Col1a1* gene (encoding type I collagen α1 chain). Complete Col1 deletion can be achieved with loss of *Col1a1* due to the fact that Col1α1 chain (encoded by *Col1a1* gene) is essential for Col1 triple helical trimer formation, while Col1α2 chain (encoded by *Col1a2* gene) alone cannot form Col1 trimers. Next, to investigate the functional role of Col1 in embryogenesis and organ development, we crossed *Col1a1*^*loxP/loxP*^ mice with *CMV-Cre* mice (Fig. 2a) to achieve the systemic (whole-body) deletion of Col1 (*CMV-Cre;Col1a1*^*loxP/loxP*^, referred to also as Col1a1^cmvKO^). *CMV-Cre* transgene, under the control of human cytomegalovirus (CMV) minimal promoter, ubiquitously deletes *loxP*-flanked genes in all tissues including germline cells. Live progeny of Col1a1^cmvKO^ was not obtained, indicating the embryonic lethality (Fig. 2b). To further determine the stage of lethality, we dissected embryos at E9.5 and E12.5 (Fig. 2c–e). Specifically, Col1a1^cmvKO^ embryos at E9.5 revealed a retarded development with the morphology that was normally observed at the E6-E7 gastrulation stage (Fig. 2c). Our results indicate the essential role of Col1 during gastrulation, consistent with previous observations identifying Col1 protein between embryonic endoderm and mesoderm^2^. Moreover, our observations that the whole-body KO of Col1 (truncated *Col1a1* exons 2-5 by CMV-Cre) resulted in failed gastrulation, arrested embryonic development at E6-E7, and the death of embryos (Fig. 2d, e), validated previous observations with a mouse model harboring the loss of Col1 by insertional mutation of *Col1a1* gene, resulting in embryonic lethality at E12-E14 due to blood vessel rupture^2^. *CMV-Cre;Col1a1*^*loxP/+*^ mice harboring heterozygous *Col1a1* loss revealed minimal abnormalities in their general health, overall survival, or their breeding ability until 6-month age (Supplementary Fig. 3a). Mild spontaneous bone fractures could be observed by X-ray examination in 6-month-old *CMV-Cre;Col1a1*^*loxP/+*^ mice (Supplementary Fig. 3b), consistent with previous studies using Osteogenesis Imperfecta type I mouse models harboring *Col1a1* heterozygous truncation/mutation^14,19^. Further investigations will be required to evaluate the mechanism by which such *Col1a1* haploinsufficiency in *CMV-Cre;Col1a1*^*loxP/+*^ mice may lead to the phenotype similar to human type I Osteogenesis Imperfecta (OI).

### Specific deletion of Col1 in Fap^+^ cells leads to impaired skeletal development and embryonic lethality

**Fig. 3:**
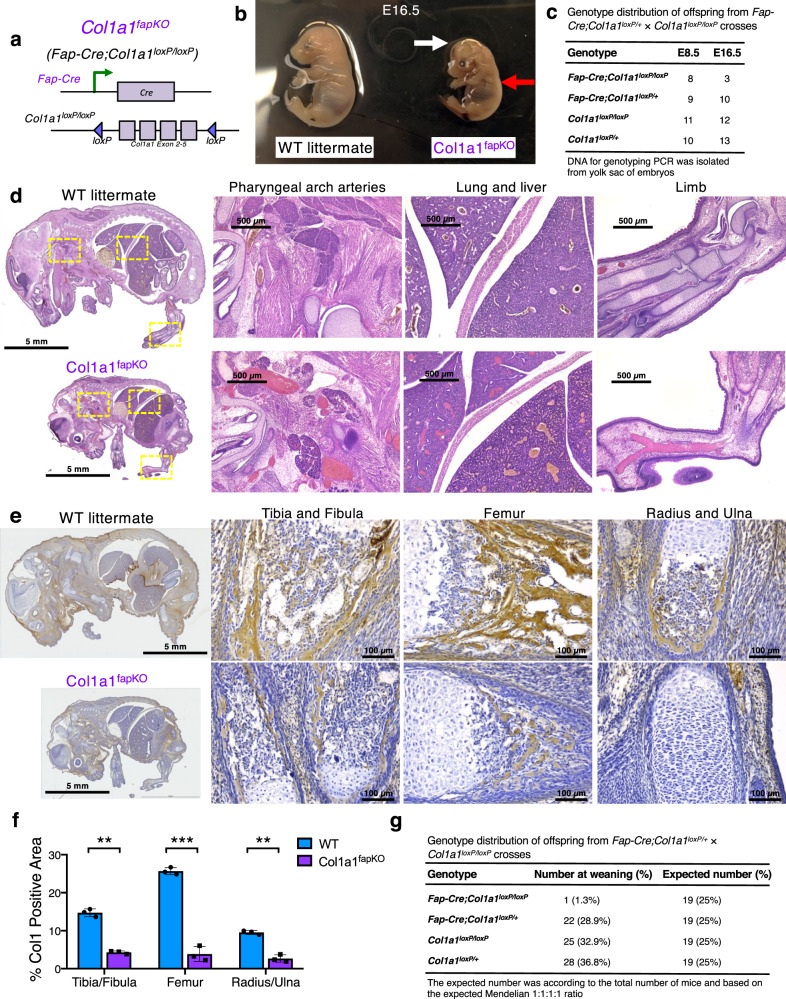
Specific deletion of Col1 in Fap-lineage cells leads to impaired skeletal development and late embryonic lethality. **a** Genetic strategy to delete *Col1a1* specifically in Fap-lineage cells by crossing the *Col1a1*^*loxP/loxP*^ mice with *Fap-Cre* mice. **b** Wild-type (WT; with genotype of *Fap-Cre-negative;Col1a1*^*loxP/loxP*^) and Col1a1^fapKO^ (*Fap-Cre;Col1a1*^*loxP/loxP*^) embryos at E16.5. The Col1a1^fapKO^ embryo exhibits hydrops fetalis (white arrow) and hemorrhage (red arrow). **c** Genotype distribution in live offspring documented examined at E9.5 and E12.5 from the crosses between *Fap-Cre;Col1a1*^*loxP/+*^ and *Col1a1*^*loxP/loxP*^ mice. **d**–**f** H&E staining (**d**) and Col1 immunohistochemistry staining (**e**) of the embryos of WT and Col1a1^fapKO^ at E16.5. Quantification of % positive area for Col1 staining was based on three mice per group (**f**). The unpaired, two-tailed *t* test was used to compare the mean of two independent groups. **P = 0.00116 (tibia/fibula), ***P = 0.00057 (femur), **P = 0.00149 (radius/ulna). Data are represented as mean ± SEM. Additional Picrosirius Red staining was shown in Supplementary Fig. 4. **g** Genotype distribution in live offspring documented at the time of weaning from the crosses between *Fap-Cre;Col1a1*^*loxP/+*^ and *Col1a1*^*loxP/loxP*^ mice. Scale bars of whole-mount sections, 5 mm; Scale bars at 50× magnification, 500 μm; Scale bars at 200× magnification, 100 μm.

To test the functional role of Col1 produced by the Fap^+^ cells in the bone, we generated the *Fap-Cre;Col1a1*^*loxP/loxP*^ (Col1a1^fapKO^) mice (Fig. 3a). Most Col1a1^fapKO^ embryos were non-viable past E16.5 and revealed prominent hemorrhage and edema (Fig. 3b), while the number of Col1a1^fapKO^ embryos at E8.5 was comparable to the expected number based on Mendelian ratio (Fig. 3c). Histological analysis further confirmed the hemorrhagic phenotype in major organs and pharyngeal arch arteries in Col1a1^fapKO^ embryos (Fig. 3d). Significantly decreased collagen deposition in skeletal system was also observed in Col1a1^fapKO^ embryos, as examined by Col1 immunohistochemical staining (Fig. 3e, f) as well as Picrosirius Red staining (Supplementary Fig. 4 and 5a). Despite the decreased Col1 deposition, the skeletal morphology of Col1a1^fapKO^ embryos did not reveal prominent deformity as compared with WT embryos. Live progeny of Col1a1^fapKO^ genotype was almost never observed with only one exception, indicating that Col1a1^fapKO^ leads to embryonic lethality with almost 100% penetrance (Fig. 3g).

**Fig. 4:**
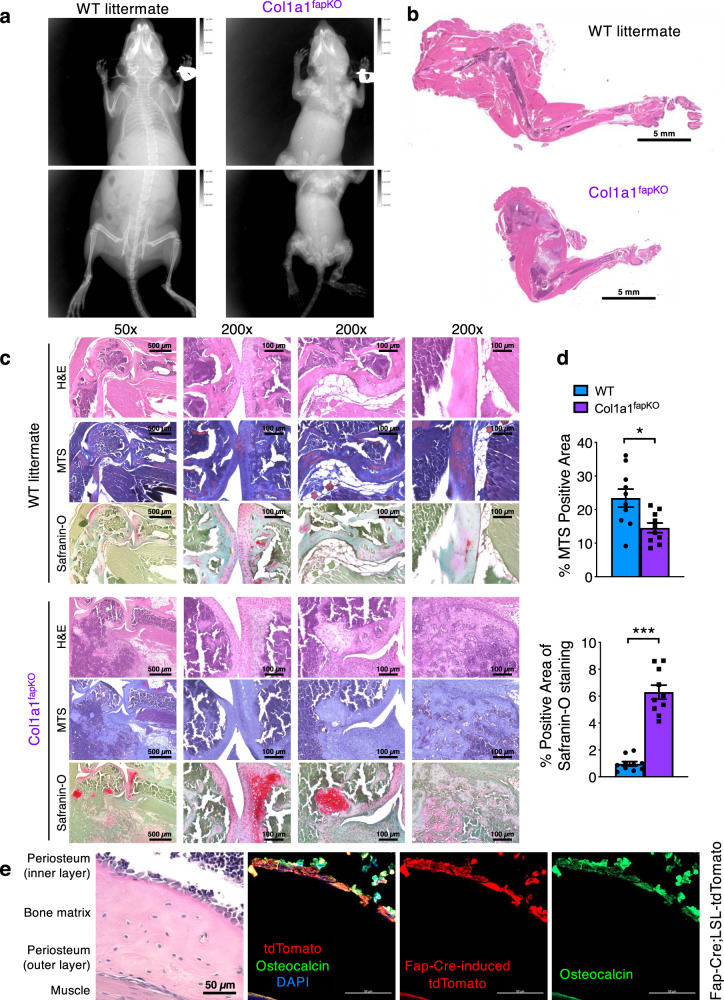
The only live offspring of Col1a1fapKO genotype exhibits severe skeletal defects. **a** Radiograph of three-week-old wild-type (WT, *Fap-Cre-negative;Col1a1*^*loxP/loxP*^) and Col1a1^fapKO^ mice. **b** H&E staining of the forelimb whole-mount sections of 3-week-old WT (*n* = 3) and Col1a1^fapKO^ (*n* = 1, limited by the fact that only one live Col1a1^fapKO^ mouse was ever observed) mice. **c** H&E staining, Masson’s Trichrome staining (MTS), and Safranin-O/Fast Green staining on serial sections of the forelimbs from three-week-old WT (*n* = 3) and Col1a1^fapKO^ (*n* = 1, limited by the fact that only one live Col1a1^fapKO^ mouse was ever observed) mice. **d** Quantification of % positive area for MTS staining (blue indicating collagen deposition) and Safranin-O/Fast Green staining (red indicating cartilage) based on the staining shown in (**c**). Quantification of % positive area for staining was based on 10 bone samples from WT group (*n* = 3 mice) and Col1a1^fapKO^ group (*n* = 1 mouse, limited by the fact that only one live Col1a1^fapKO^ mouse was ever observed). The unpaired, two-tailed *t* test was used to compare the mean of two independent groups. *P = 0.0116, ***P = 1.11E-06. Data are represented as mean ± SEM. **e** Representative H&E and immunofluorescence images of humerus serial sections from *Fap-Cre;LSL-tdTomato* mice (*n* = 3 mice at 2-month-old) stained for osteoblast marker osteocalcin (green) and *Fap-Cre*-induced tdTomato (red). Scale bars, 50 μm. Scale bars of whole-mount sections, 5 mm; Scale bars at 50× magnification, 500 μm; Scale bars at 200× magnification, 100 μm.

Despite the fact that most Col1a1^fapKO^ embryos died prenatally, we indeed observed one live progeny with Col1a1^fapKO^ genotype. This mouse was drastically smaller in size compared to its littermates with severely defective/fractured skeleton throughout the body (Fig. 4a). Histological analysis further revealed the dramatic fractures in Col1a1^fapKO^ mouse (Fig. 4b), associated with significantly decreased Col1 deposition in the bones (Fig. 4c, d). The fractures in Col1a1^fapKO^ mouse was accompanied by the significant accumulation of chondrocytes and cartilage deposition, as examined by H&E and Safranin-O/Fast Green staining (Fig. 4c, d). Prominent deformities were also observed in both the limb skeleton and the axial skeleton of the Col1a1^fapKO^ mouse (Supplementary Fig. 5b). A lineage-tracing *Fap-Cre;LSL-tdTomato* mouse model exhibited colocalization between *Fap-Cre*-tdTomato^+^ cells and osteoblast marker osteocalcin (Fig. 4e), consistent with previous sc-RNA-seq analysis showing *Fap*-expression in osteolineage cells (Fig. 1e). These results do not rule out the possibility that Fap^+^ fibroblasts may derive from osteolineage cells or osteoblasts. Taken together, these results demonstrate the fundamental role of Fap^+^ cells in the de novo production of Col1in vascular organogenesis and bone development during embryogenesis.

### Specific deletion of Col1 in Fsp1^+^ cells leads to Osteogenesis Imperfecta phenotype in mice

**Fig. 5:**
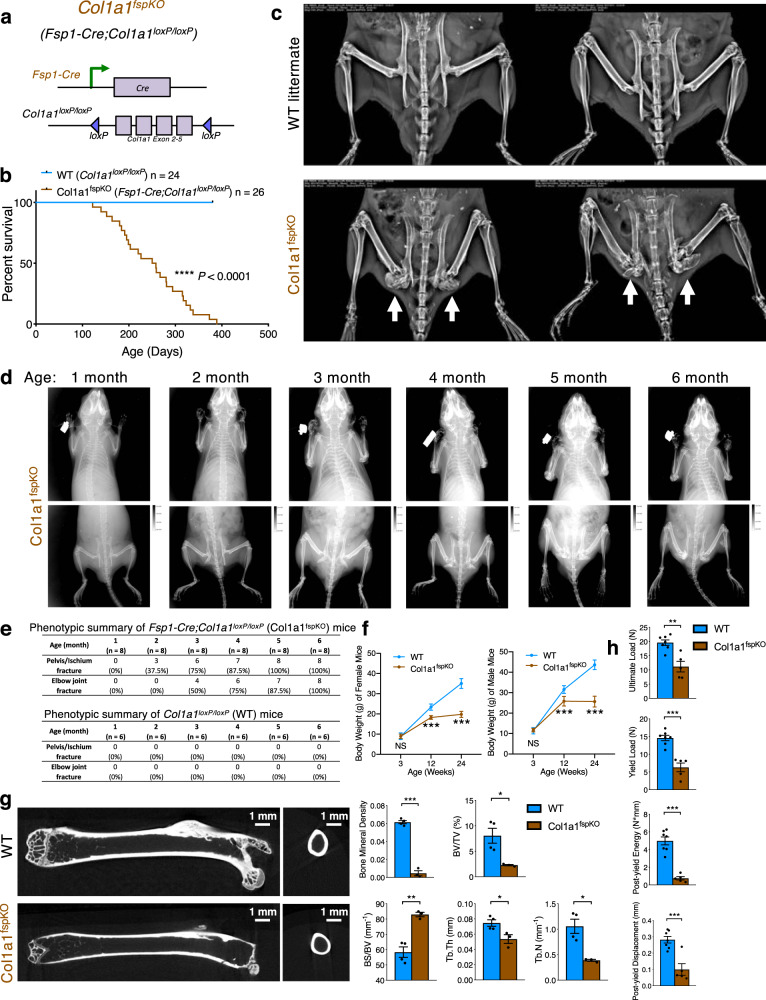
Specific deletion of Col1 in Fsp1-lineage cells causes Osteogenesis Imperfecta phenotype. **a** Genetic strategy to delete *Col1a1* specifically in Fsp1-lineage cells by crossing the *Col1a1*^*loxP/loxP*^ mice with *Fsp1-Cre* mice. **b** Survival of wild-type (WT; with genotype of *Fsp1-Cre-negative;Col1a1*^*loxP/loxP*^) and Col1a1^fspKO^ (*Fsp1-Cre;Col1a1*^*loxP/loxP*^) mice (*n* = 24 and 26, respectively). Kaplan-Meier plots and the log rank Mantel-Cox test were used to evaluate statistical differences of survival. ****P < 0.0001. **c** Micro-CT scanning of 6-month-old WT and Col1a1^fspKO^ mice (with white arrows indicating the fractures in pelvis and ischium of Col1a1^fspKO^ mice). **d** Radiograph of Col1a1^fspKO^ mice at the age from 1 to 6 month. Radiograph of age-matched WT littermates was shown in Supplementary Fig. 7a. **e** Summary of the occurrence of fractures in pelvis, ischium, and/or joints, as examined by radiograph, of WT (*n* = 6) and Col1a1^fspKO^ (*n* = 8) mice at the age from 1 to 6 month. **f** Body weight of WT and Col1a1^fspKO^ mice (*n* = 6 mice per group) at the age of 3, 12, and 24 month. The unpaired, two-tailed *t* test was used to compare the mean of two independent groups. ***P = 9.57E-05 (female 12 weeks), 7.15E-07 (female 24 weeks), 0.00096 (male 12 weeks), 2.35E-07 (male 24 weeks). Data are represented as mean ± SEM. **g** The micro-CT images of trabecular and cortical bones of femurs from 6-month-old WT mice (*n* = 4) and Col1a1^fspKO^ mice (*n* = 3). The trabecular bone material property measurements are compared as bone mineral density (BMD; ***P = 1.04E-05), bone volume/total volume (BV/TV; * *P* = 0.0289), bone surface area/bone volume (BS/BV; **P = 0.00212), trabecular thickness (Tb.Th; *P = 0.0287), and trabecular number (Tb.N; *P = 0.0160). The unpaired, two-tailed *t* test was used to compare the mean of two independent groups. Data are represented as mean ± SEM. The cortical bone material property measurements are continued in Supplementary Fig. 7d. **h** Biomechanical (three-point bending) test results of femurs from 6-month-old WT mice (*n* = 7) and Col1a1^fspKO^ mice (*n* = 5) were presented as ultimate load (**P = 0.00135), yield load (***P = 0.00012), post-yield energy (***P = 2.01E-05), and post-yield displacement (***P = 0.00077). The unpaired, two-tailed *t* test was used to compare the mean of two independent groups. Data are represented as mean ± SEM.

To test the function of Col1 produced by the Fsp1^+^ cells in the bone stroma, we generated the *Fsp1-Cre;Col1a1*^*loxP/loxP*^ (Col1a1^fspKO^) mice (Fig. 5a). These mice were born normal but Col1 deletion in Fsp1-lineage cells led to the occurrence of spontaneous Osteogenesis Imperfecta (OI) symptoms in adult Col1a1^fspKO^ mice, which was analogous to the previous observations employing systemic transgenic mice harboring dominant mutations of *Col1a1* and *Col1a2*^6,12,14,15,38,39^. Col1a1^fspKO^ mice developed OI phenotype with full penetrance, resulting in death of mice at around 6-12 months of age (Fig. 5b). At the age of 6 month, all Col1a1^fspKO^ mice exhibited the inevitable occurrence of fractures and deformations, frequently observed in the joints, pelvis, ischium, and head of femur (Fig. 5c, d). Such OI-like phenotype of Col1a1^fspKO^ mice was very similar to the well-characterized *Col1a2*^*OIM/OIM*^ mice^6^ and phenotypically resembled the moderate to severe OI type III in humans. As shown in Supplementary Fig. 6a, b as a reference, the *Col1a2*^*OIM/OIM*^ mouse model harbors homozygous whole-body point-mutated *Col1a2* alleles and develops severe OI feature in the bones, similar to the Col1a1^fspKO^ mice.

Further radiographic analysis revealed that mild deformation of pelvis and ischium was observed as early as 2–3 months of age, and more severe fracture in hindlimbs associated with dysfunction by the 5–8 months or age (Fig. 5d, e). Noticeable deformation of elbow joints can also be seen as early as 3–4 months of age, resulting in forelimb dysfunction by 6-9 months of age (Fig. 5d, e). In contrast, the age-matched wild-type (*Fsp1-Cre-negative;Col1a1*^*loxP/loxP*^) littermates did not reveal any of the above symptoms (Supplementary Fig. 7a). Of note, Col1a1^fspKO^ mice were born at the expected Mendelian ratio (Supplementary Fig. 7b). Additionally, Col1a1^fspKO^ mice exhibited no gross physical or behavioral abnormalities before one-month of age (Fig. 5e, f). Significant body weight loss and hampered movement were observed in all Col1a1^fspKO^ mice by 3–6 months of age (Fig. 5f and Supplementary Movie 1). Female and male Col1a1^fspKO^ mice revealed similar body weight loss and overall survival (Fig. 5f and Supplementary Fig. 7c). Micro-CT measurements confirmed the significantly decreased bone mineral density and bone material properties in six-month-old Col1a1^fspKO^ mice as compared with age-matched WT littermates (Fig. 5g and Supplementary Fig. 7d). Spontaneous fractures in the head of femur were ubiquitously observed in Col1a1^fspKO^ mice (Fig. 5g). Biomechanical assays validated the brittleness of femurs from six-month-old Col1a1^fspKO^ mice as compared with WT littermates (Fig. 5h).

**Fig. 6:**
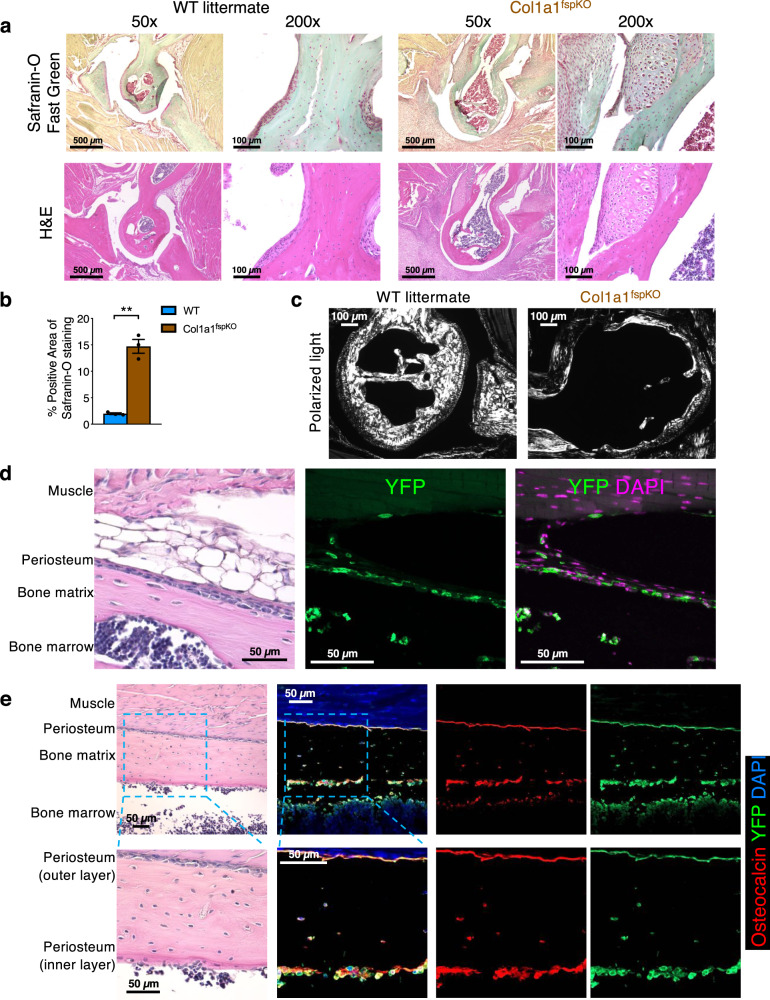
Histological analysis of bone tissues from Col1a1fspKO mice and Fsp1-Cre;LSL-YFP mice. **a**, **b** H&E staining and Safranin-O/Fast Green staining of the forelimb elbow joints of 6-month-old WT and Col1a1^fspKO^ mice (**a**). Quantification of % positive area for Safranin-O/Fast Green staining was based on three mice per group (**b**). The unpaired, two-tailed *t* test was used to compare the mean of two independent groups. **P = 0.00946. Data are represented as mean ± SEM. Scale bars at 50× magnification, 500 μm; Scale bars at 200× magnification, 100 μm. **c** Picrosirius Red staining of collagen content in the forelimb elbow joints from six-month-old WT and Col1a1^fspKO^ mice (*n* = 3 mice per group) examined by circularly polarized light microscopy. Scale bars, 100 μm. **d** Representative immunofluorescence images of humerus serial sections from lineage-tracing *Fsp1-Cre;LSL-YFP* mice (*n* = 3 mice at two-month-old) stained for *Fsp1-Cre*-induced YFP. Scale bars, 50 μm. **e** Representative H&E and immunofluorescence images of humerus serial sections from *Fsp1-Cre;LSL-YFP* mice (*n* = 3 mice at two-month-old) stained for osteoblast marker osteocalcin (red) and *Fsp1-Cre*-induced YFP (green). Scale bars, 50 μm.

Next, we performed histological analysis of joint lesions from Col1a1^fspKO^ mice (Fig. 6a and Supplementary Fig. 7e). In the joint lesions with fractures and deformations, we observed significant callus formation composed of chondrocytes and cartilage components adjacent to the fractured areas, as observed by Safranin-O staining of cartilage matrix (Fig. 6a, b). The joint lesions from Col1a1^fspKO^ mice also revealed significantly decreased overall levels of fibrillar Col1, as examined using circularly polarized light microscopy on Picrosirius Red-stained sections (Fig. 6c). Lineage-tracing *Fsp1-Cre;LSL-YFP* mouse model revealed *Fsp1-Cre*-induced YFP expression in the periosteum, connective tissue, and bone marrow (Fig. 6d). *Fsp1-Cre*-induced YFP expression was also observed in osteocalcin-expressing osteoblasts in a mosaic pattern (Fig. 6e). These observations indicate that the phenotypes of Col1a1^fspKO^ mice may result from the combined effect of Col1 deletion in Fsp1 cell lineage including both osteoblasts and other periosteal cells. Collectively, these results establish Col1a1^fspKO^ mice as a novel cell-specific transgenic mouse model for OI. Our observations that the Col1a1^fspKO^ mice reveal insignificant abnormalities in the bones before 1 month of age and develop OI symptoms after 2–3 months of age, emphasize the crucial role of Col1 produced specifically by Fsp1-lineage cells in regulating proper bone development and healing of fractures.

### Col1 deletion in Fsp1^+^ cells alters the composition of neutrophils and macrophages in the bone marrow

**Fig. 7:**
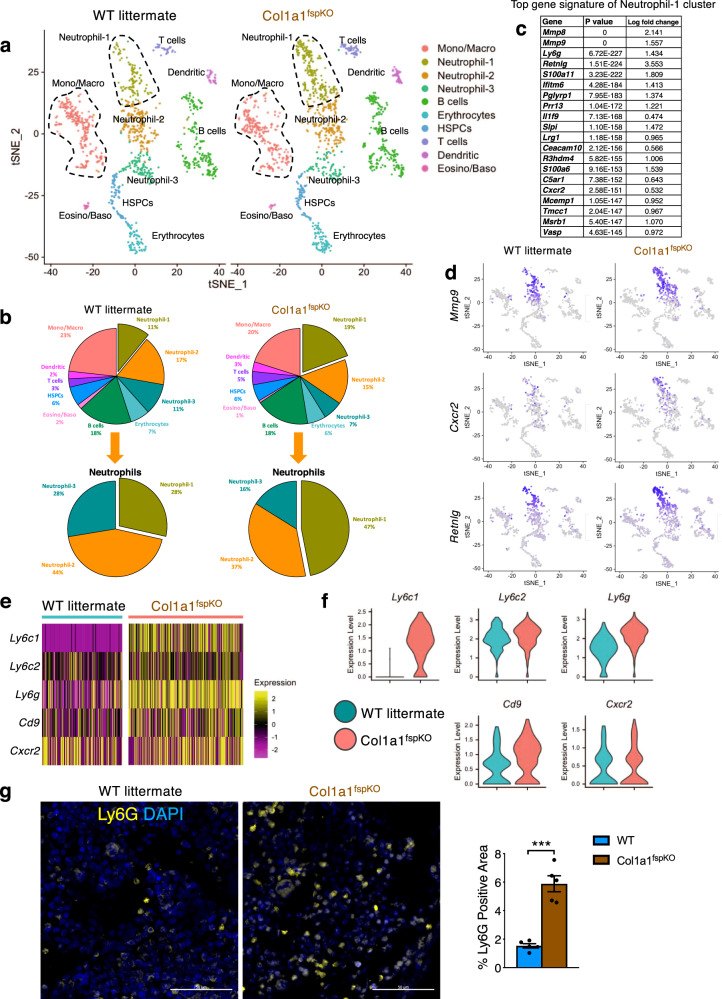
Single-cell RNA-sequencing analysis of bone marrow fractions from Col1a1fspKO mice. **a**–**c** Single-cell RNA-sequencing analysis of cell mixture of bone marrow fractions from 2-month-old WT and Col1a1^fspKO^ mice (*n* = 2 per group). Functional clusters of cells were defined with group definition listed, as implemented in the Seurat R package (**a**). Mono/Macro: monocyte/macrophage cluster. HSPCs: hematopoietic stem and progenitor cells. Dendritic: dendritic cell cluster. Eosino/Baso: eosinophil/basophil cluster. **b** Pie charts showing the percentage of each of the functional cell clusters in bone marrow fractions from WT and Col1a1^fspKO^ mice. Total neutrophil clusters containing 3 subpopulations (1–3) were further plotted as pie charts. (**c**) List of top signature genes of “Neutrophil-1” cluster shown in (**a**) and (**b**), presented with *P* value (based on the non-parameteric Wilcoxon Rank Sum test) and Log2 fold change. Continued in Supplementary Fig. 8a–c. **d** Expression profile of *Mmp9*, *Cxcl12*, and *Retnlg* among defined cell clusters in the bone marrow fractions from WT and Col1a1^fspKO^ mice shown in UMAP plot. Continued in Supplementary Fig. 8d. **e**, **f** Top upregulated genes of Neutrophil-1 cluster in Col1a1^fspKO^ mice than WT mice shown in heat map plot (**e**) or violin plot (**f**). **g** Representative immunofluorescence images and quantification results of humerus bone marrow from two-month-old WT and Col1a1^fspKO^ mice (*n* = 5 per group) stained for Neutrophil-1 marker Ly6G (yellow). Scale bars, 50 μm. The unpaired, two-tailed *t* test was used to compare the mean of two independent groups. ***P = 0.00096. Data are represented as mean ± SEM.

In order to further investigate the impact of Col1 deletion in the Fsp1^+^ cells on the bone marrow microenvironment, we conducted single-cell RNA-sequencing analysis (sc-RNA-seq) on unfractionated live cell mixture from Col1a1^fspKO^ mouse bone marrow (Fig. 7a, Supplementary Fig. 8a–c). Col1a1^fspKO^ mouse bone marrow was examined at 2-month age (when OI phenotype initiates in these mice), as compared with age-matched wild-type littermate bone marrow. Most of the major cell lineages (such as T cell, B cell, erythroid cell, eosinophil/basophil, and dendritic cell populations) remained largely unchanged in Col1a1^fspKO^ mouse bone marrow (Fig. 7a, b). Interestingly, Col1a1^fspKO^ mouse bone marrow revealed significantly increased percentage of a specific neutrophil cluster (“Neutrophil-1” cluster, Fig. 7a, b) when compared to wild-type littermate bone marrow. This uniquely increased neutrophil cluster revealed high expression of matrix metalloproteinase 9 (MMP9) (Fig. 7c, d), which has been previously shown to regulate bone fracture repair^26,40,41^. This MMP9-high Neutrophil-1 subpopulation highly expressed *Mmp8*, *Cxcr2*, and *Retnlg* (Fig. 7c, d, and Supplementary Fig. 8d, e), as well as the general neutrophil markers such as *S100a8*, *Ly6g*, and *Itgam*/CD11b (Supplementary Fig. 8d). The top upregulated genes in the Neutrophil-1 cluster of Col1a1^fspKO^ bone marrow (in comparison with WT bone marrow) were largely anti-inflammatory neutrophil marker genes including *Ly6g*, *Ly6c1*, *Ly6c2*, and *Cxcr2* (Fig. 7e, f). The significant enrichment of Ly6G^+^ neutrophils in Col1a1^fspKO^ bone marrow was also confirmed by immunofluorescence staining (Fig. 7g). Ingenuity Pathway Analysis (IPA) of signature genes in the Neutrophil-1 cluster (Supplementary Fig. 8e) revealed the correlation with neutrophil degranulation/activation. Additionally, the monocyte/macrophage cluster of Col1a1^fspKO^ mouse bone marrow also revealed a noticeable shift towards Adgre1 (F4/80)-expressing subpopulation (Supplementary Fig. 8f), while the overall percentage of monocyte/macrophage cluster exhibiting a mild increase.

In conclusion, these results suggest an involvement of neutrophils and monocytes/macrophages in response to the bone fractures during early OI pathogenesis in Col1a1^fspKO^ mice^42–46^.

### Normal phenotype of mice with specific deletion of Col1 in αSMA^+^ mesenchymal cells or Cdh5^+^ endothelial cells

Additionally, we also generated the *αSMA-Cre;Col1a1*^*loxP/loxP*^ (Col1a1^smaKO^) and *Cdh5-Cre;Col1a1*^*loxP/loxP*^ (Col1a1^cdh5KO^) mouse models, with the potential to delete Col1 in αSMA^+^ mesenchymal cells and Cdh5(VE-Cadherin)^+^ endothelial cells, respectively. Both Col1a1^smaKO^ and Col1a1^cdh5KO^ mice were born at the expected Mendelian ratio, when compared to their littermate controls (Supplementary Fig. 9a, b). These mice exhibit normal tissue throughout the body, normal litter size, and unaltered life span, without any overt phenotype. Fractures were not observed in either Col1a1^smaKO^ or Col1a1^cdh5KO^ mice at 12-month age (Supplementary Fig. 9c, d). The body weight of Col1a1^smaKO^ or Col1a1^cdh5KO^ mice at 12-month age was identical to that of their wild-type littermates (Supplementary Fig. 9e, f). These results were not surprising, given that αSMA^+^ mesenchymal cell populations and Cdh5^+^ endothelial cells in the bones showed minimal *Col1a1* expression (Fig. 1d). These results indicate that the contribution of αSMA^+^ cells and Cdh5^+^ cells to Col1 production in the bones is not rate limiting. Although Col1a1^smaKO^ mice did not reveal spontaneous bone fractures, profound accumulation of αSMA-expressing cells in periosteal area was observed (Supplementary Fig. 9g) in the spontaneous bone lesions of Col1a1^fspKO^ mice, consistent with previous studies showing the involvement in fracture repair. Future studies will hopefully unravel whether Col1a1^smaKO^ mice may exhibit any bone healing deficiency^47–50^.

## Discussion

Type I collagen (Col1) is the most abundant protein in the human body, especially in the bones. Over 25,000 publication have examined the role of Col1 in different aspects of biology in health and disease, but it remained unknown until this study what cellular lineages contribute to the synthesis and deposition of Col1 during embryogenesis and bone development^3,4^. Osteoblasts and fibroblasts, differentiated from mesenchymal stem/stromal cells (MSCs), are speculated to be the major source of Col1 in various organs. However, the precise contributions of Col1 by different cell lineages in the formation and development of bones remain unknown. In this regard, previous dataset^27^ coupled with current analysis show that Col1 is predominantly produced by Fap^+^ and Fsp1^+^ mesenchymal cells in the bone. Based on this data, we generated novel genetically engineered mouse models (GEMMs) for the deletion of Col1 specifically in Fap^+^ or Fsp1^+^ cells. A *Col1a1*^*loxP/loxP*^ mouse strain was generated and crossed with *CMV-Cre*, *Fap-Cre*, *Fsp1 (S100a4)-Cre*, *Cdh5-Cre*, and *αSMA (Acta2)-Cre* mice to probe the origin and function of Col1 during embryogenesis and bone development.

Our results demonstrate that systemic (whole-body) deletion of *Col1a1* (exons 2–5 truncation) employing *CMV-Cre* mice (Col1a1^cmvKO^) leads to arrested embryonic development and embryonic lethality around embryonic day 6–7 (E6-7), underscoring the essential role of Col1 during gastrulation stage. Deletion of Col1 in Fap^+^ cells leads to embryonic lethality at around E16 associated with several skeletal and vascular defects. This observation is in alliance with previous studies with Fap^+^ cells identified as mesenchymal cells surrounding the vasculature and in the skeletal structures^51,52^. These observations suggest the essential role of Fap^+^ cells in the synthesis of Col1 during the embryonic development of the bones. The observed phenotype of Col1a1^fapKO^ mice is analogous to the human type II Osteogenesis Imperfecta, which is lethal in the perinatal period due to critical genetic defects in *COL1A1* or *COL1A2* resulting in multiple rib and/or long bone fractures^53–55^.

Many different studies identified Fsp1^+^ cells in the developing skeletal tissue during embryogenesis^35,56,57^. Specific deletion of Col1 in Fsp1^+^ cells leads to an Osteogenesis Imperfecta-like phenotype and with most Col1a1^fspKO^ mice dying at the age of 5–12 months. The phenotype of Col1a1^fspKO^ mice is comparable to the human type III Osteogenesis Imperfecta, and relevant mouse models previously reported^6,13–15,19,38,58–60^. While previous models of OI were based on systemic Col1 mutation and/or deletion, our studies identity the precise Fsp1 cell lineage that is responsible for Col1 production in maturing bones starting about three weeks of age, and absence of such synthesis by the Fsp1^+^ cells leads to abnormal bones, resulting in easy fractures with minimal trauma. Our studies collectively suggest that early skeletal development during embryogenesis and the early postnatal period might rely on Fap^+^ cells for the production of Col1, and at a later stage the role of Fsp1^+^ cells becomes prominent. Deletion of Col1 from αSMA^+^ cells did not result in any adversity and mice were normal with expected lifespan. Same is true when Col1 was deleted in Cdh5^+^ endothelial cells (a common biomarker for most endothelial cells). This experimental arm served as an important control, because some reports suggest that angiogenic endothelial cells during embryogenesis and organogenesis are shown to produce type I collagen. All together, these experiments suggest that αSMA^+^ mesenchymal cells do not compensate for the loss of Col1 from Fap or Fsp1 cell lineages. Moreover, the functions of Fap^+^ cells and Fsp1^+^ cells are mutually exclusive in the context of embryogenesis and bone formation.

These results also emphasize the crucial contribution of Col1 specifically produced by Fsp1^+^ osteoblasts and fibroblasts (in bone marrow and periosteum) to the structural maintenance and repair of the bones, which is consistent with previous notion that fibroblasts appear at fracture site and are responsible for collagen production/crosslinking upon encountering hematoma^61^. Our results also suggest that the Col1 deposited by fibroblasts of Fsp1-lineage is essential for the formation of hematoma, which further impacts the subsequent steps leading to the repair of fractures including osteoblast differentiation and bone formation in the fractured callus^62–64^. The identification of the Fsp1^+^ cells as critical and rate-limiting source of Col1 in developing bone and adult bone undergoing repair and regeneration provides new insights into potential cell-based therapy for Osteogenesis Imperfecta. Future studies may examine whether Fsp1^+^ cells may provide cell-based therapy opportunities for Osteogenesis Imperfecta via transplantation.

Taken together, our results demonstrate that Col1 produced by Fap^+^ cells is essential for the embryonic development of skeletal system, while Col1 produced by Fsp1^+^ cells is critical for the postnatal maintenance and repair of the bones. Utilizing multiple transgenic mouse models and single-cell RNA-sequencing analyses, this study for the first time provided comprehensive investigations on the functional roles of Col1 with respect to its specific cellular origin during embryogenesis, osteogenesis, and bone pathogenesis.

## Methods

### Mice

All mice were housed under standard housing conditions at MDACC animal facilities, and all animal procedures were reviewed and approved by the MDACC Institutional Animal Care and Use Committee.

*Fsp1-Cre*^65,66^ and *αSMA-Cre*^67^ mouse strains were previously documented. *Cdh5-Cre* (017968; B6.129-Tg-Cdh5-cre-1Spe/J), *CMV-Cre* (006054; B6.C-Tg-CMV-cre-1Cgn/J), and *LSL-YFP* (006148; B6.129 × 1-Gt-ROSA-26Sortm1-EYFP-Cos/J) mouse strains were purchased from Jackson Laboratory. Osteogenesis imperfecta murine (OIM) strain harboring *Col1a2* mutation (001815; B6C3Fe *a*/*a*-*Col1a2oim*/J) was purchased from Jackson Laboratory. This *Col1a2* spontaneous mutation is a single nucleotide deletion (G) that alters the terminal approximately 50 amino acids of the pro-alpha 2 C-propeptide and prevents association with the pro-alpha1 chains. *Col1a1*^*loxP/loxP*^ mouse strain (with *loxP*-flanked exons 2-5) was established in the Genetically Engineered Mouse Facility at MD Anderson Cancer Center (MDACC) using the Col1a1^tm1a(EUCOMM)Wtsi^ embryonic stem cells that were obtained from the European Mouse Mutant Cell Repository (EuMMCR)^68^. Both female and male mice with desired genotype(s) were used for experimental mice.

### Genotyping

Tail DNA was used for genotyping PCR analysis of animals and embryos older than E14.5. Yolk sac DNA was used for genotyping PCR of embryos at earlier stages. Recombination PCR of *Col1a1*^*loxP/loxP*^ allele on DNA from multiple major organs of all experimental mice was routinely performed to exclude the possibility of germline recombination.

### Histology and immunohistochemistry

For paraffin-fixed samples, mouse tissues were fixed in 10% neutral buffered formalin, embedded in paraffin, and sectioned at 5 μm thickness. Sections were processed for hematoxylin and eosin (H&E) staining. Masson’s trichrome stain (MTS) was conducted using Gomori’s Trichrome Stain Kit (38016SS2, Leica Biosystems). Picrosirius red staining for collagen was conducted using 0.1% Picrosirius Red (Direct Red80; Sigma-Aldrich) and counterstained with Weigert’s haematoxylin. Images were captured with a Leica DM 1000 LED microscope and an MC120 HD Microscope Camera with LAS V4.4 Software (Leica). Safranin-O/Fast Green staining was conducted using 0.1% Safranin-O solution and 0.1% Fast Green solution (Electron Microscopy Sciences).

Formalin-fixed, paraffin-embedded sections were processed for immunohistochemical staining as previously documented^69,70^. Sections were incubated with primary antibody against type I collagen (Col1; SouthernBiotech, 1310-01, lot#B2918-XB89, 1:200), followed by biotinylated donkey anti-goat secondary antibody (Jackson ImmunoResearch, 705-066-147, lot#127411, 1:400), and streptavidin HRP (Biocare Medical). For all immunolabeling experiments, sections were developed by DAB and counterstained with hematoxylin. Immunofluorescence staining on formalin-fixed, paraffin-embedded sections was conducted using the following primary antibodies: rabbit anti-GFP/YFP (Abcam, ab290, lot#GR3321614-1, 1:500), chicken anti-GFP/YFP (Abcam, ab13970, lot# GR3190550-13, 1:400), rabbit anti-osteocalcin/OCN (Millipore/Sigma-Aldrich, ab10911, lot#3670782, 1:200), rat anti-Ly6G (Abcam, ab25377, clone RB6-8C5, lot#GR3391587-2, 1:100), and mouse anti-RFP/tdTomato (Thermo Fisher, MA5-15257, clone RF5R, lot#SD247965, 1:100). The staining was then followed by AF488/594-labeled secondary antibodies: Thermo Fisher A11012 (lot#1933366), A11039 (lot#2079383), A11008 (lot#1937184), and A21203 (lot#1722995), all in 1:400 dilution. Slides were then mounted with DAPI-containing Vectashield Mounting Medium (Vector Laboratories), visualized under the LSM800 confocal laser scanning microscope, and analyzed with ZEN software version 2.1 (Zeiss).

### Radiography (X-ray) and micro computed tomography (micro-CT) Scanning

Imaging operations were conducted at MDACC Small Animal Imaging Facility. Radiography of mice was conducted using the Bruker Xtreme system. Micro-CT scanning was performed using the Bruker SkyScan 1276 micro-CT system (Bruker BioSpin Corporation). The scanning parameters are: resolution: 13 μm; pixel size: 13.083 μm; voltage: 55 kV; current: 200 μA; rotation step (deg): 0.400; duration: 11 min; exposure time: 500 ms; filter: 0.25 mm Al; matrix: 2016 ×1344. The femurs of 6-month-old Col1a1^fspKO^ mice and wild-type (WT) littermates were scanned and measured for the bone material properties. The trabecular bone parameters were measured in the distal metaphysis of the femurs, while the cortical bone parameters were measured at the femur mid-diaphysis. The detected bone parameters included trabecular or cortical bone mineral density (BMD), bone volume/total volume (BV/TV), trabecular or cortical bone surface area/bone volume (BS/BV), trabecular thickness (Tb.Th), trabecular number (Tb.N), cortical bone total area (Tt.Ar), cortical bone area (Ct.Ar), cortical bone marrow area (Ma.Ar),), cortical bone area/total area (Ct.Ar/Tt.Ar), and cortical thickness (Ct.Th). The analysis was conducted using Bruker SkyScan DataViewer software version 1.5.0.

### Biomechanical (three-point bending) examination

The femurs from Col1a1^fspKO^ mice and WT littermates of 6-month age were freshly collected and tested at room temperature using an Instron 5848 microtester (Instron Inc.), as previously documented^17^. The femurs were examined to failure in three-point bending test at a rate of 0.1 mm/s. A 100 N load cell was used to collect data. Load and displacement data were captured at a rate of 40 Hz by Bluehill Software version 2.9 (Instron). The span length used in the examination was 7.3 mm. The maximum load was determined by the highest load value recorded before the specimen fractured.

### Single-cell RNA-sequencing (sc-RNA-seq)

For the sc-RNA-seq on bone marrow cells conducted in this study, femur and tibia bones of Col1a1^fspKO^ mice (and age-matched wild-type littermate; *n* = 2 mice per group) were collected and flushed to obtain bone marrow fractions. Bone marrow cells, pooled for each group, were filtered by 70 μm cell strainers, and resuspended in PBS/2%FBS as single cell suspension. The single cell suspension was stained with Live/Dead viability dye eFluor 780 (65-0865-14, eBioscience), filtered through a 40 μm mesh, and then sorted for live cells with Aria II sorter (BD Biosciences) at the South Campus Flow Cytometry Core Laboratory of MDACC. These samples were examined by sc-RNA-seq at the Sequencing and Microarray Facility of MDACC. Single cell Gel Bead-In-Emulsions (GEMs) generation and barcoding, post GEM-RT cleanup and cDNA amplification, library construction and Illumina-ready sequencing library generation were prepared by following the manufacturer’s guidelines. High Sensitivity dsDNA Qubit kit was used to estimate the cDNA and Library concentration. HS DNA Bioanalyzer was used for the quantification of cDNA. DNA 1000 Bioanalyzer was used for the quantification of libraries. The “c-loupe” files were generated by using Cell Ranger software pipelines following manufacturer’s guidelines. Cells from unfractionated tumor were encapsulated using 10X Genomics’ Chromium controller and Single Cell 3’ Reagent Kits v2. Following capture and lysis, cDNA was synthesized and amplified to construct Illumina sequencing libraries. The libraries from about 1,000 cells per sample were sequenced with Illumina Nextseq 500. The run format was 26 cycles for read 1, 8 cycles index 1, and 124 cycles for read 2. sc-RNA-seq data was processed by the Sequencing and Microarray Facility in MD Anderson Cancer Center. Library Seurat (version 3.6.1), dplyr (version 1.0.7) and cowplot (version 1.1.1) were loaded into R (version 3.6.1) to explore QC metrics, filter cells, normalize data, cluster cells, and identify cluster biomarkers. To filter out low-quality cells, a threshold with a minimum of 200 and a maximum of 7000 genes per cell was used. Cells with more than 10% of the mitochondrial genome were also removed for further analysis. “RunTSNE” function was used for clustering the cells. Based on the “JackStrawPlot” and “ElbowPlot” functions, the first 27 principal components were used for TSNE projection and clustering analysis. “FindAllMarkers” function was used to identify the specific markers for each cell cluster. “DoHeatmap” function was used to show the top 10 genes in each cluster. “VlnPlot” function was used to show expression probability distributions across cell clusters of the genes we selected to assign the cell type identity, and the genes that we were interested in.

The previous published single-cell RNA-sequencing (sc-RNA-seq) dataset on bone and bone marrow cell populations of healthy mice^27^ were retrieved from the Gene Expression Omnibus (GEO) repository (GSE128423). As previously described^27^, femur and tibia bones of healthy C57BL/6 mice were collected and separated into bone and bone marrow fractions. Stromal cells from the bone marrow were isolated after STEMxyme1/DispaseII digestion. Stromal cells from the bone fraction were isolated after STEMxyme1/DispaseII digestion of the cut/crushed bone fragments. Cell mixtures were then filtered through 70 μm cell strainers, lysed with ACK-lysis buffer, and prepared for FACS cell sorting. Bone marrow stromal cells were enriched by sorting of live cells that are negative for erythroid markers (CD71/Ter119) and immune lineage markers (CD45/CD3/B220/CD19/Gr-1/CD11b). This dataset was analyzed using a similar methodology as the above sc-RNA-seq data generated in this study. A total of 12530 cells from bone fraction and 14875 cells from bone marrow fraction were analyzed. Functional clusters of cells were defined with indicated group definition, as implemented in the Seurat R package. “RunUMAP” function was used for clustering the cells. “FindAllMarkers” function was used to identify the specific markers for each cell cluster. “FindMarkers” function was used to compare the differentially expressed marker genes for indicated cell cluster between control and knockout groups. “DoHeatmap” function was used to show the top 10 genes in each cluster. “VlnPlot” function was used to show expression probability distributions across cell clusters of the genes we selected to assign the cell type identity, and the genes that we were interested in.

### Statistics

Statistical analyses were performed with unpaired, two-tailed *t* test, or one-way ANOVA with Tukey’s multiple comparison test using GraphPad Prism version 8.0.0 (GraphPad Software, San Diego, CA, USA). Kaplan–Meier plots were drawn for survival analysis and the log rank Mantel-Cox test was used to evaluate statistical differences. Data met the assumptions of each statistical test, where variance was not equal (determined by an *F*-test) Welch’s correction for unequal variances was applied. A *P* value <0.05 was considered statistically significant. *P < 0.05, **P < 0.01, ***P < 0.001, ****P < 0.0001, NS not significant. Error bars represented standard error of the mean (S.E.M.) when multiple visual fields were averaged to produce a single value for each animal, which was then averaged again to represent the mean bar for the group in each graph.

### Reporting summary

Further information on research design is available in the Nature Research Reporting Summary linked to this article.

## Supplementary information

Supplementary Information

Reporting Summary

Description of Additional Supplementary Files

Supplementary Movie 1

## Data Availability

The authors declare that all data supporting the findings of this study are available within the paper and the supplementary information. The single-cell RNA-sequencing source data generated by this study have been deposited in Gene Expression Omnibus (GEO) database with the accession number GSE184360. The single-cell RNA-sequencing source data of a previously published dataset^27^ have been deposited in Gene Expression Omnibus (GEO) database with the accession number GEO GSE128423. Source data are provided with this paper.

## Source data

Source Data
