# Supplementary material for: Type-I collagen produced by distinct fibroblast lineages reveals specific function during embryogenesis and Osteogenesis Imperfecta
Source: Nat Commun. 2021 Dec 10;12:7199. doi: 10.1038/s41467-021-27563-3 (PMC8664945; doi:10.1038/s41467-021-27563-3)
Supplement: Supplementary file 1 — Supplementary Information [file 41467_2021_27563_MOESM1_ESM.pdf]

Supplementary Figure 1

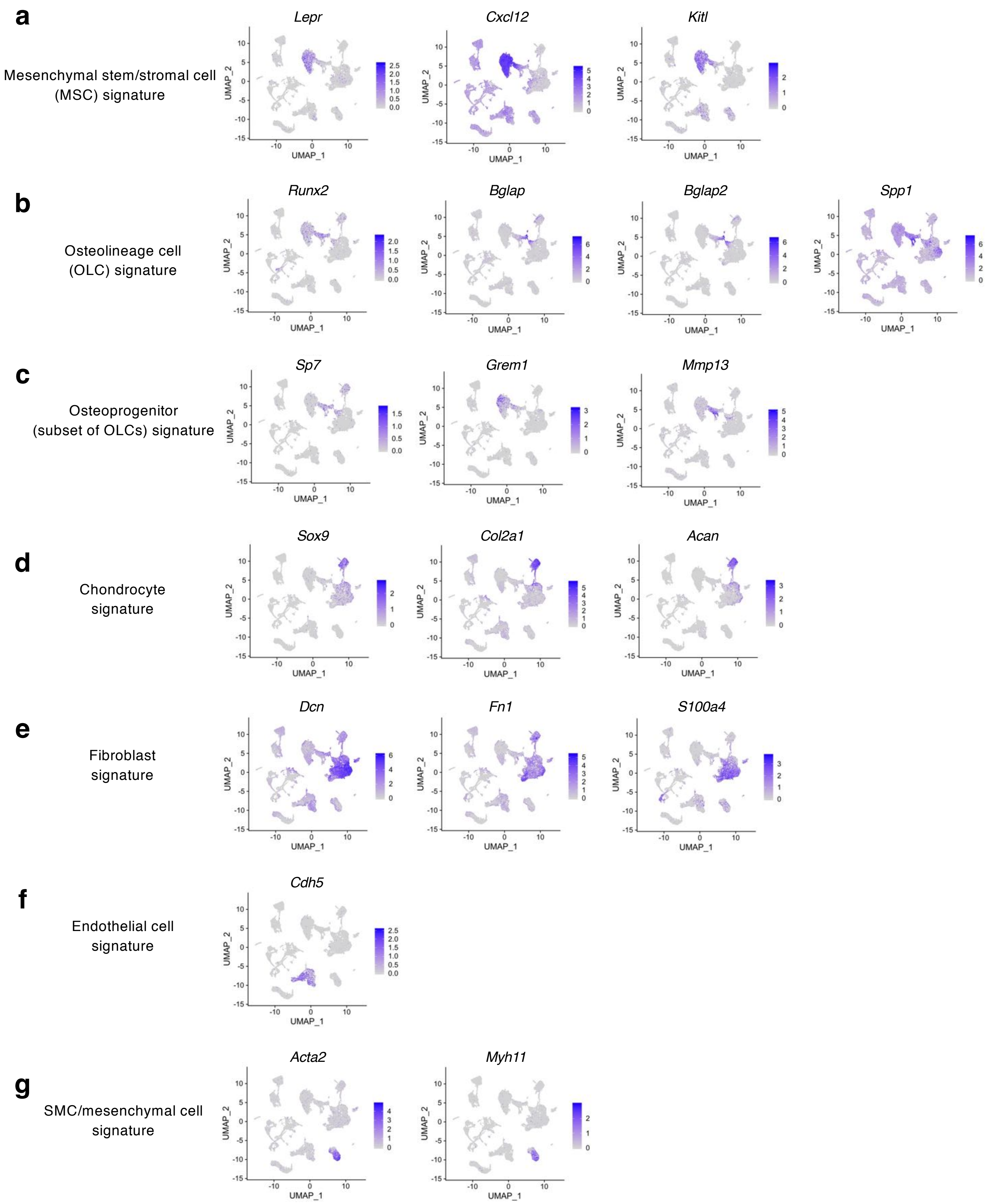

Supplementary Fig. 1. Single-cell RNA-sequencing analysis of non-hematopoietic cells from mouse bone and bone marrow fractions

a-g Single-cell RNA-sequencing analysis of cell mixture of mouse bone and bone marrow fractions continued from **Fig. 1**. Expression profile of mesenchymal stem/stromal cell (MSC) markers: *Lepr*, *Cxcl12*, and *Kitl* (**a**); MSC-descendent osteolineage cell (OLC) markers: *Runx2*, *Bglap, Bglap2,* and *Spp1* (**b**); osteoprogenitor cell markers: *Sp7*, *Grem1*, and *Mmp13* (**c**); chondrocyte markers: *Sox9*, *Acan*, and *Col2a1* (**d**); fibroblast markers: *Dcn*, *Fn1*, and *S100a4* (**e**); bone marrow endothelial cell (BMEC) marker: *Cdh5* (**f**); and SMC/mesenchymal cell markers: *Acta2* and *Myh11* (**g**), among defined cell clusters shown in UMAP plot.

Supplementary Figure 2

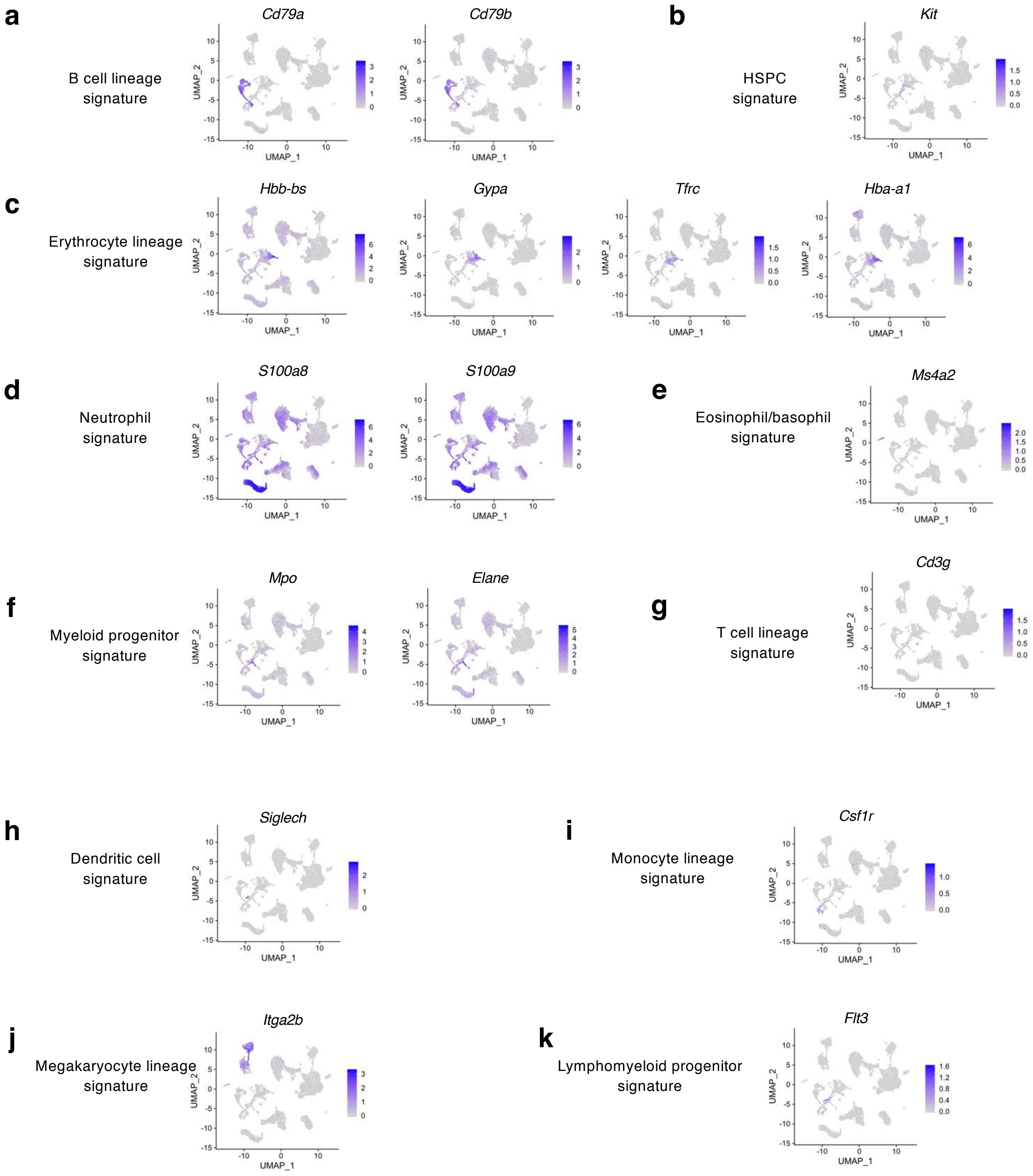

Supplementary Fig. 2. Single-cell RNA-sequencing analysis of hematopoietic cells from mouse bone and bone marrow fractions

a-k Single-cell RNA-sequencing analysis of cell mixture of mouse bone and bone marrow fractions continued from **Fig. 1**. Expression profile of B cell lineage markers: *Cd79a* and *Cd79b* (**a**); Hematopoietic stem and progenitor cell (HSPC) marker: *Kit* (**b**); erythrocyte lineage markers: *Hbb-bs*, *Gypa*, *Tfrc* (CD71), and *Hba-a1* (**c**); neutrophil markers: *S100a8* and *S100a9* (**d**); eosinophil/basophil marker: *Ms4a2* (**e**); myeloid progenitor cell markers: *Mpo* and *Elane* (**f**); T cell lineage marker: *Cd3g* (**g**); dendritic cell marker: *Siglech* (**h**); monocyte cell marker: *Csf1r* (**i**); megakaryocyte lineage marker: *Itga2b* (**j**); and lymphomyeloid progenitor marker: *Flt3* (**k**), among defined cell clusters shown in UMAP plot.

Supplementary Figure 3

a

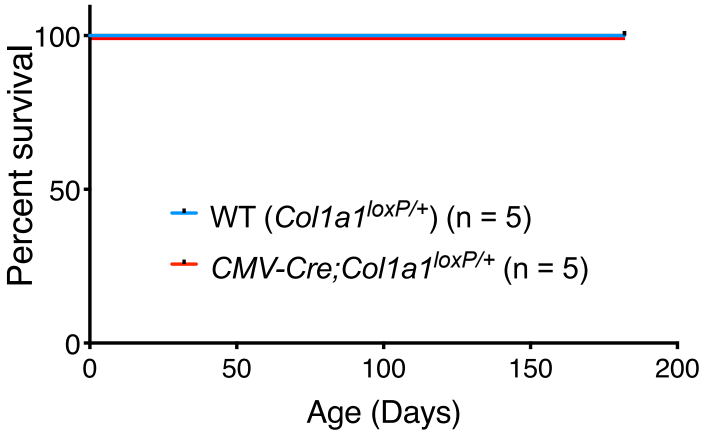

b

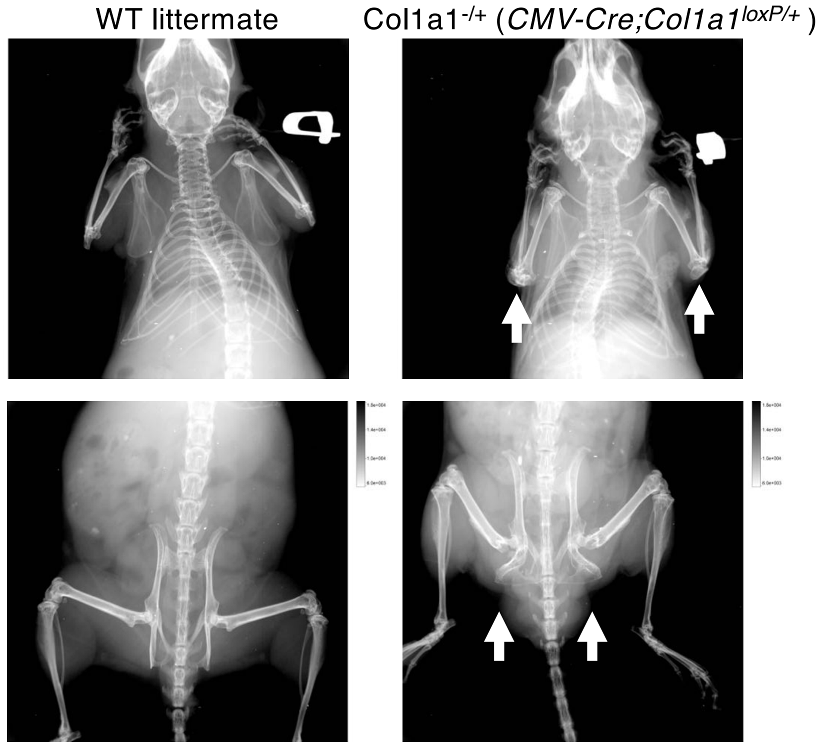

Supplementary Fig. 3. Characterization of *CMV-Cre;Col1a1*$^{loxP/+}$ mice with *Col1a1* haploinsufficiency

a Overall survival of *CMV-Cre;Col1a1*$^{loxP/+}$ mice harboring heterozygous *Col1a1* loss, in comparison with age-matched wild-type (WT; *Cre-negative; Col1a1*$^{loxP/+}$) littermates (n = 5 per group) observed for 6 months.

b Representative radiographic images of 6-month-old *CMV-Cre;Col1a1*$^{loxP/+}$ mice, in comparison with age-matched wild-type littermates. Arrows indicate spontaneous fracture lesions in elbow joint and pelvis of *CMV-Cre;Col1a1*$^{loxP/+}$ mice.

Supplementary Figure 4

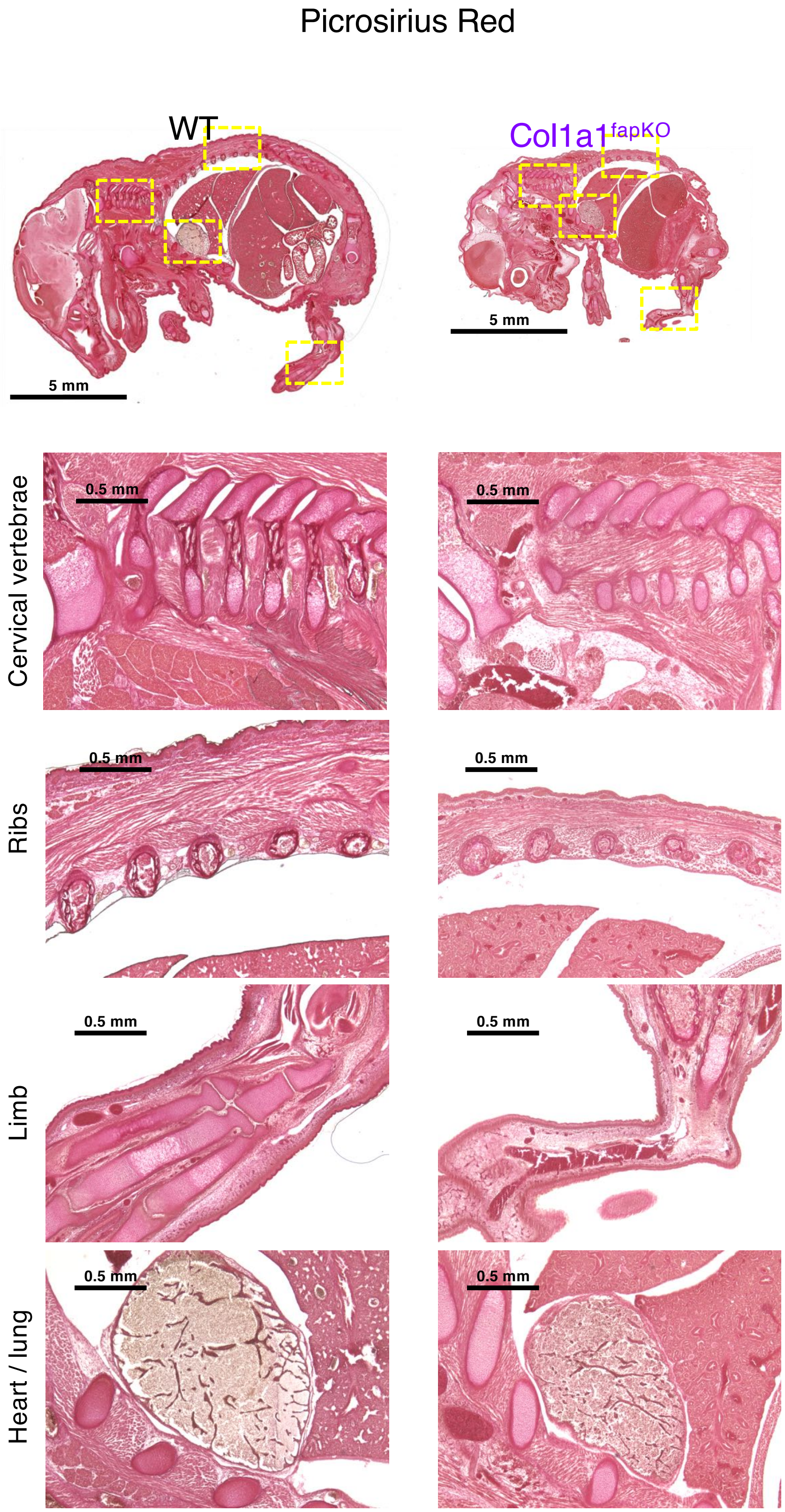

Supplementary Fig. 4. Specific deletion of Col1 in Fap-lineage cells leads to impaired collagen deposition in the bones

Picrosirius Red staining of the embryos of wild-type (WT; *Fap-Cre-negative;Col1a1*$^{loxP/loxP}$) and Col1a1^{fapKO} (*Fap-Cre;Col1a1*$^{loxP/loxP}$) at E16.5, continued from **Fig. 3e**. Representative images were shown for n = 4 mice per group. Scale bars of whole-mount sections, 5 mm; Scale bars at 50× magnification, 0.5 mm.

Supplementary Figure 5

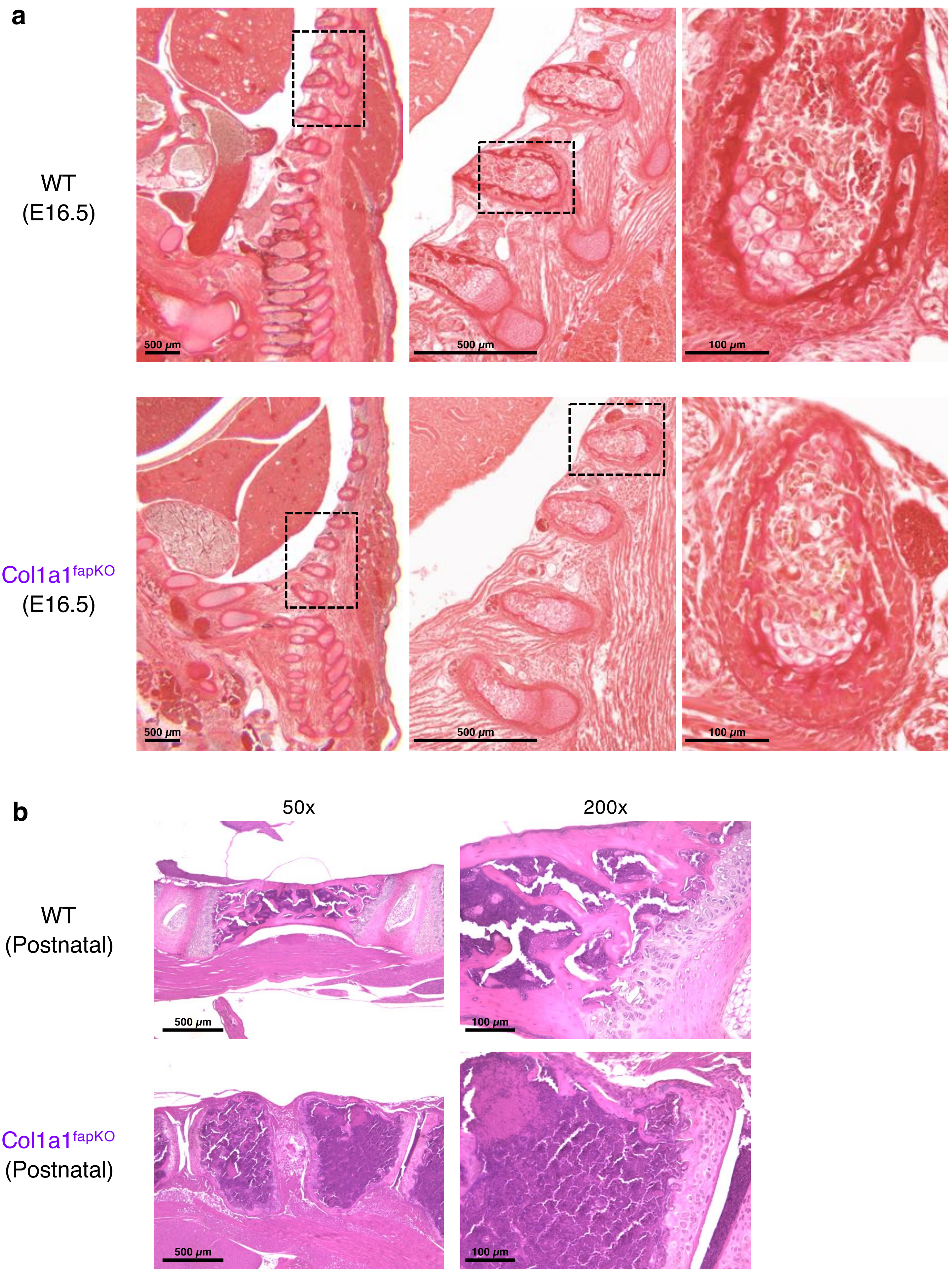

Supplementary Fig. 5. Col1 deletion in Fap-lineage cells results in defective vertebrate development

a Picrosirius Red staining of the vertebrates in E16.5 embryos from Col1a1^{fapKO} (*Fap-Cre;Col1a1*$^{loxP/loxP}$) and WT groups (continued from **Supplementary Fig. 4**). Representative images were shown for n = 3 embryos per group. Scale bars at 50×/100x magnification, 0.5 mm. Scale bars at 400× magnification, 0.1 mm.

b Representative H&E staining images of the vertebrates from the only live Col1a1^{fapKO} mouse (n = 1 mouse, limited by the fact that only one live Col1a1^{fapKO} mouse was ever observed) and its WT littermate (n = 3 mice). Scale bars at 50× magnification, 0.5 mm. Scale bars at 200× magnification, 0.1 mm.

Supplementary Figure 6

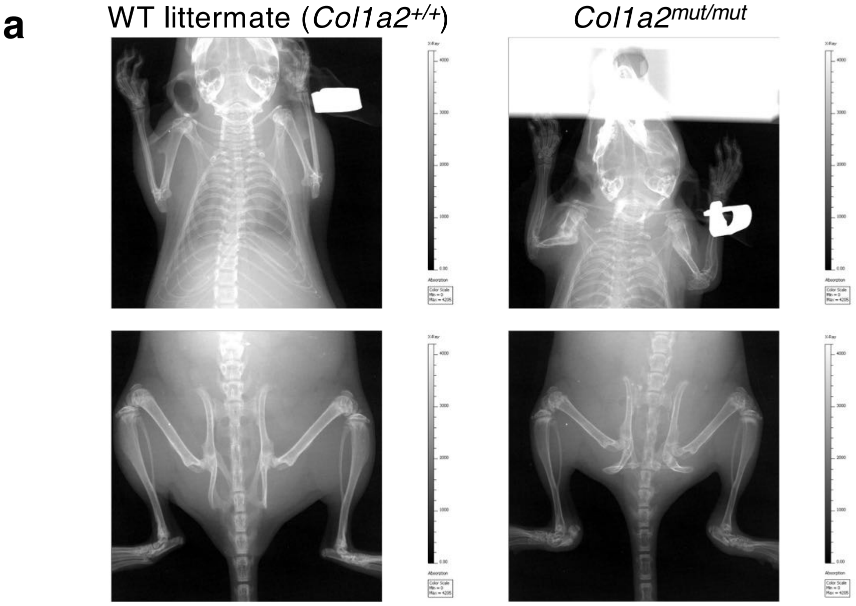

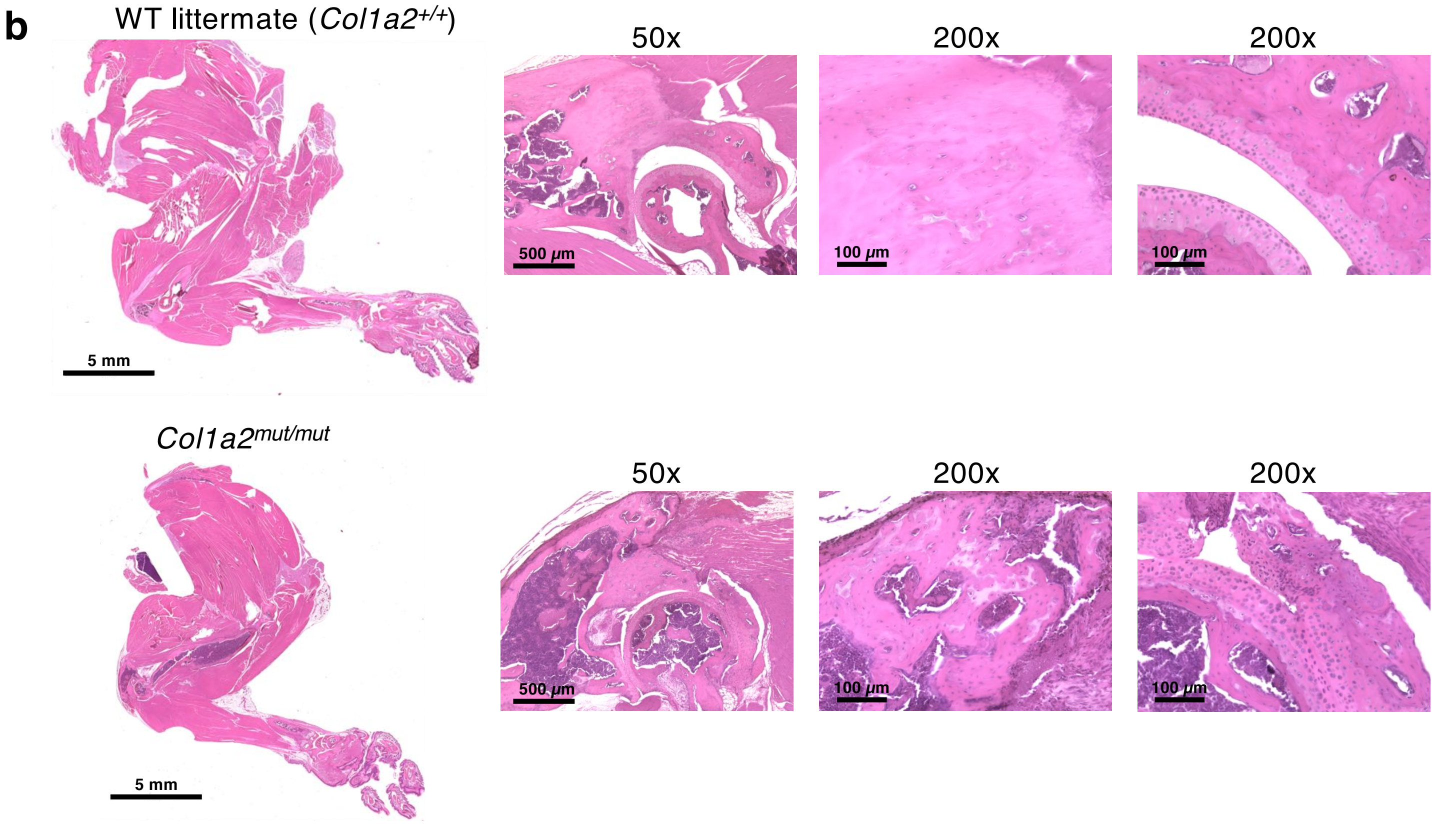

Supplementary Fig. 6. *Col1a2$^{OIM/OIM}$* mice exhibit similar Osteogenesis Imperfecta phenotype to Col1a1^{fspKO} mice

a Radiograph of 2.5-month-old wild-type (WT; *Col1a2$^{+/+}$*) and OIM (*Col1a2$^{OIM/OIM}$*) mice.

b H&E staining of the forelimb sections of 2.5-month-old WT and OIM mice. Representative images were shown for n = 6 mice per group. Scale bars of whole-mount sections, 5 mm; Scale bars at 50× magnification, 500 μm; Scale bars at 200× magnification, 100 μm.

Supplementary Figure 7

a

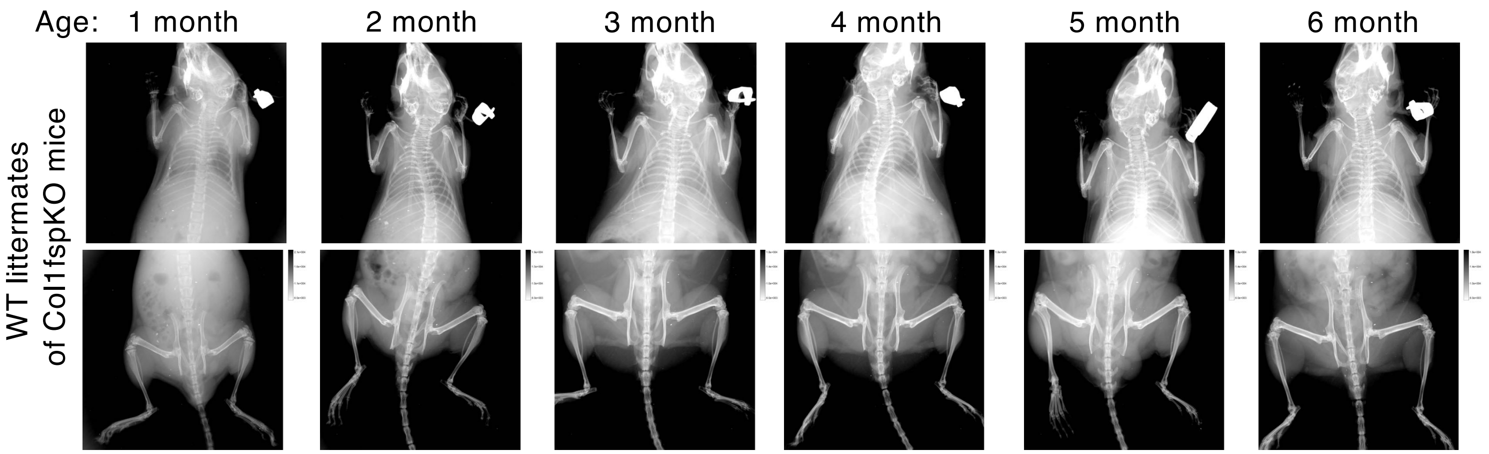

b

Genotype distribution of offspring from *Fsp1-Cre;Col1a1*$^{loxP/loxP}$ × *Col1a1*$^{loxP/loxP}$ crosses

Genotype	Number at weaning (%)	Expected number (%)
Fsp1-Cre;Col1a1$^{loxP/loxP}$	51 (47%)	54.5 (50%)
Col1a1$^{loxP/loxP}$	58 (53%)	54.5 (50%)

The expected number was according to the total number of mice and based on the expected Mendelian 1:1 ratio

c

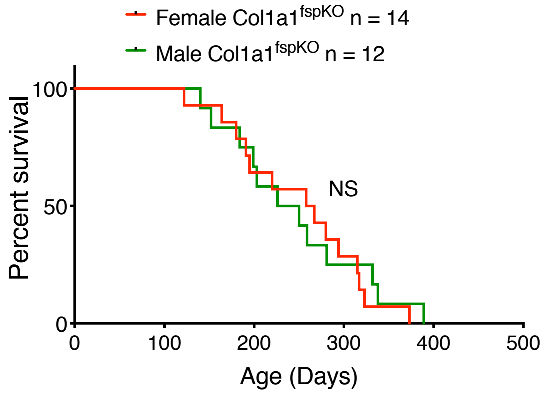

d

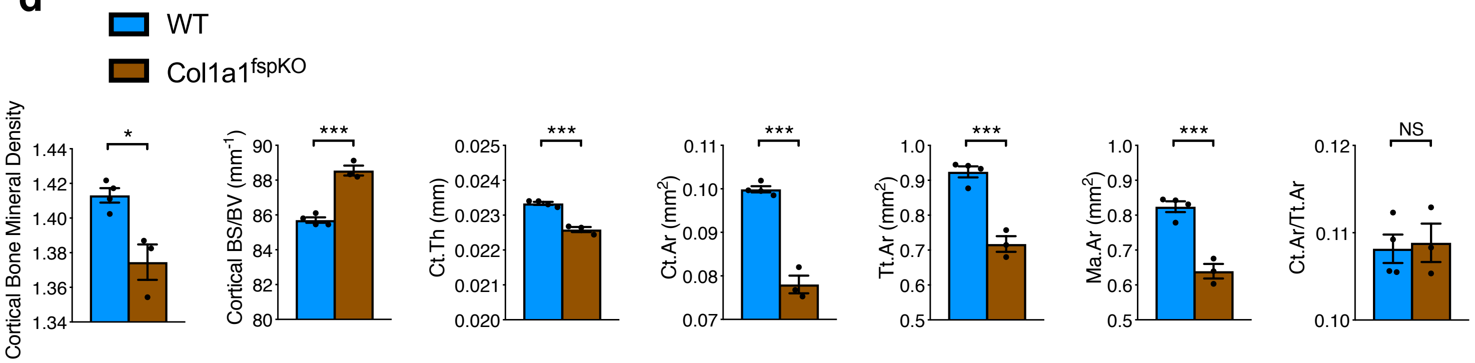

e

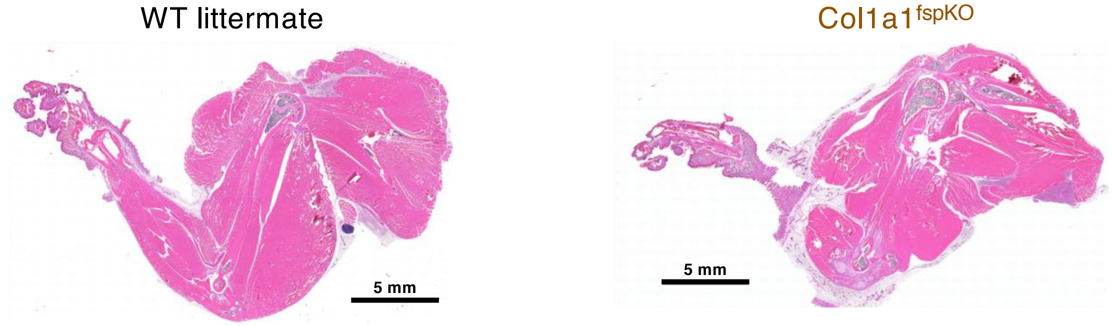

Supplementary Fig. 7. Col1 deletion in Fsp1-lineage cells results in frequent spontaneous fractures of the bones

a Radiograph of WT (*Fsp1-Cre-negative;Col1a1*$^{loxP/loxP}$) mice at the age from 1 to 6 month. These WT mice are age-matched littermates of Col1a1^{fspKO} (*Fsp1-Cre;Col1a1*$^{loxP/loxP}$) mice shown in **Fig. 5d**.

b Genotype distribution in live offspring documented at the time of weaning from the crosses between *Fsp1-Cre;Col1a1*$^{loxP/loxP}$ and *Col1a1*$^{loxP/loxP}$ mice.

c Survival of female (n = 14) and male (n = 10) mice of Col1a1^{fspKO} (*Fsp1-Cre;Col1a1*$^{loxP/loxP}$) genotype, based on the survival data of the same mouse cohort originally shown in **Fig. 5b**. Kaplan-Meier plots and the log rank Mantel-Cox test were used to evaluate statistical differences of survival. NS, not significant.

d Continued from **Fig. 5g**. The cortical bone material property measurements of 6-month-old WT mice (n = 4) and Col1a1^{fspKO} mice (n = 3) are compared as bone mineral density (BMD; * $P = 0.0114$), bone surface area/bone volume (BS/BV; *** $P = 0.00023$), cortical thickness (Ct.Th; *** $P = 0.00022$), cortical bone area (Ct.Ar; *** $P = 8.84E-05$), total area (Tt.Ar; *** $P = 0.00055$), cortical bone marrow area (Ma.Ar; *** $P = 0.00075$), and cortical area/total area (Ct.Ar/Tt.Ar; NS not significant). The unpaired, two-tailed *t* test was used to compare the mean of two independent groups. Data are represented as mean ± SEM.

e H&E staining of the forelimb whole-mount sections of 6-month-old WT and Col1a1^{fspKO} mice, continued from **Fig. 6a**. Scale bars, 5 mm.

Supplementary Figure 8

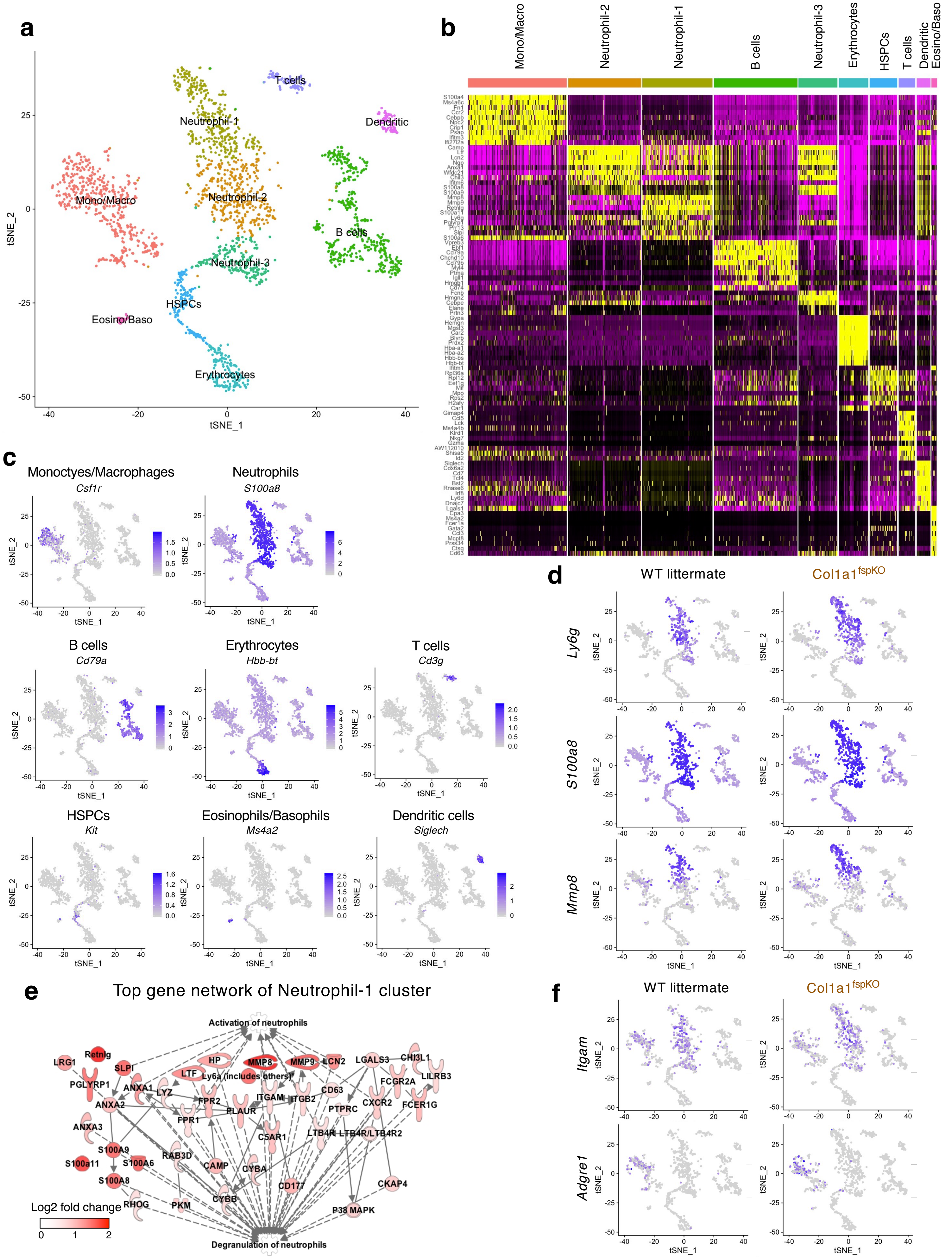

Supplementary Fig. 8. Single-cell RNA-sequencing analysis of bone marrow fractions from Col1a1^{fspKO} mice (continued from Fig. 7)

a-d Single-cell RNA-sequencing analysis of cell mixture of bone marrow fractions from 2-month-old WT and Col1a1^{fspKO} mice, originally shown in **Fig. 7**. Functional clusters of cells, combined from WT and Col1a1^{fspKO} mice, were shown in (**a**). (**b**) Heat map showing the scaled expression values of discriminating signature genes for the functional clusters of cells defined above. (**c**) Expression profile of representative signature gene for each cell cluster shown in UMAP plot. (**d**) Expression profile of *Ly6g*, *S100a8*, and *Mmp8* among defined cell clusters in the bone marrow fractions from WT and Col1a1^{fspKO} mice shown in UMAP plot.

e Ingenuity pathway analysis (IPA) visualizing the significantly upregulated signature genes (red) of "Neutrophil-1" cell cluster.

f Expression profile of *Itgam* (CD11b) and *Adgre1* (F4/80) among defined cell clusters in the bone marrow fractions from WT and Col1a1^{fspKO} mice.

Supplementary Figure 9

a

Genotype distribution of offspring from *αSMA-Cre;Col1a1*$^{loxP/loxP}$ × *Col1a1*$^{loxP/loxP}$ crosses

Genotype	Number at weaning (%)	Expected number (%)
αSMA-Cre;Col1a1$^{loxP/loxP}$	72 (48%)	75 (50%)
Col1a1$^{loxP/loxP}$	78 (52%)	75 (50%)

The expected number was according to the total number of mice and based on the expected Mendelian 1:1 ratio

b

Genotype distribution of offspring from *Cdh5-Cre;Col1a1*$^{loxP/loxP}$ × *Col1a1*$^{loxP/loxP}$ crosses

Genotype	Number at weaning (%)	Expected number (%)
Cdh5-Cre;Col1a1$^{loxP/loxP}$	15 (51.7%)	14.5 (50%)
Col1a1$^{loxP/loxP}$	14 (48.3%)	14.5 (50%)

The expected number was according to the total number of mice and based on the expected Mendelian 1:1 ratio

c

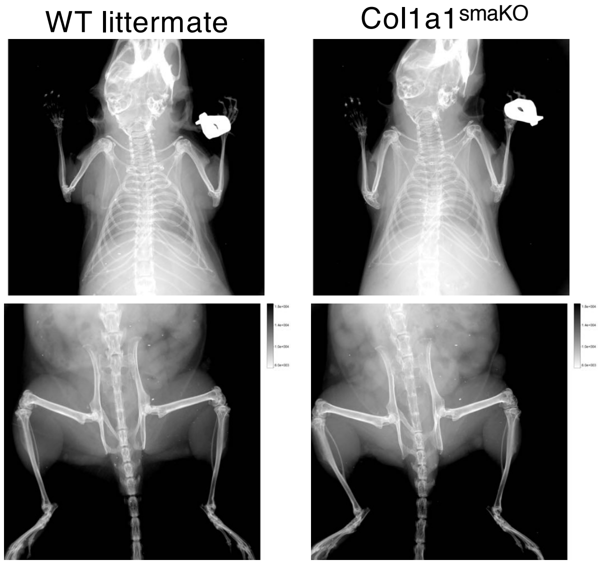

d

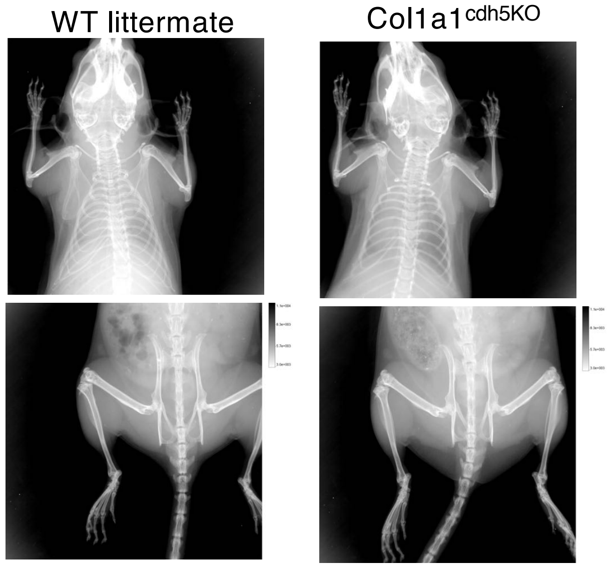

e

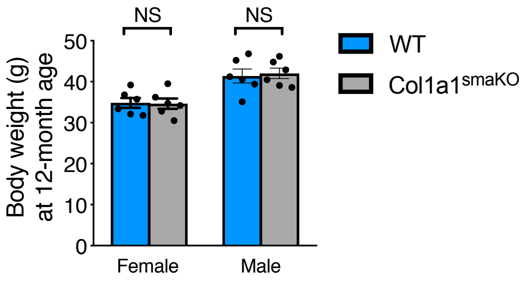

f

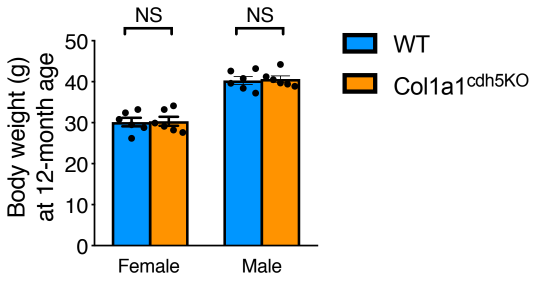

g

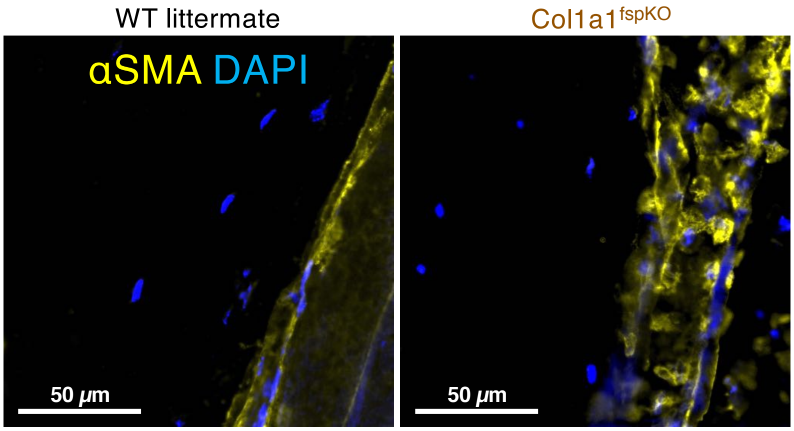

Supplementary Fig. 9. Col1 deletion in αSMA-lineage mesenchymal cells or Cdh5-lineage endothelial cells causes no abnormal phenotype in mice

a,b Genotype distribution in live offspring documented at the time of weaning from the crosses between *αSMA-Cre;Col1a1*$^{loxP/loxP}$ (Col1a1^{smaKO}) and WT (*Cre-negative;Col1a1*$^{loxP/loxP}$) mice (**a**), or *Cdh5-Cre;Col1a1*$^{loxP/loxP}$ (Col1a1^{cdh5KO}) and WT mice (**b**).

c,d Radiograph of 12-month-old WT mice, Col1a1^{smaKO} mice (**c**), and Col1a1^{cdh5KO} mice (**d**).

e,f Body weight of 12-month-old WT mice, Col1a1^{smaKO} mice (**e**), and Col1a1^{cdh5KO} mice (**f**). Comparison of mouse body weight was based on 6 mice per group. The unpaired, two-tailed *t* test was used to compare the mean of two independent groups. NS, not significant. Data are represented as mean ± SEM.

g Representative immunofluorescence images of humerus periosteal areas from 6-month-old WT and Col1a1^{fspKO} mice (n = 4 mice per group) stained for αSMA (yellow). Enrichment of αSMA-expressing cells emerged in response to spontaneous fracture lesions in Col1a1^{fspKO} mice. Scale bars, 50 µm.
