# Supplementary material for: Type-I collagen produced by distinct fibroblast lineages reveals specific function during embryogenesis and Osteogenesis Imperfecta
Source: Nat Commun. 2021 Dec 10;12:7199. doi: 10.1038/s41467-021-27563-3 (PMC8664945; doi:10.1038/s41467-021-27563-3)
Supplement: Supplementary file 2 — Reporting Summary [file 41467_2021_27563_MOESM2_ESM.pdf]

nature portfolio

Corresponding author(s): Raghu Kalluri

Last updated by author(s): Sep 25, 2021

Reporting Summary

Nature Portfolio wishes to improve the reproducibility of the work that we publish. This form provides structure for consistency and transparency in reporting. For further information on Nature Portfolio policies, see our Editorial Policies and the Editorial Policy Checklist.

Statistics

For all statistical analyses, confirm that the following items are present in the figure legend, table legend, main text, or Methods section.

n/a	Confirmed	
☐	☒	The exact sample size (*n*) for each experimental group/condition, given as a discrete number and unit of measurement
☐	☒	A statement on whether measurements were taken from distinct samples or whether the same sample was measured repeatedly
☐	☒	The statistical test(s) used AND whether they are one- or two-sided<br>*Only common tests should be described solely by name; describe more complex techniques in the Methods section.*
☐	☒	A description of all covariates tested
☒	☐	A description of any assumptions or corrections, such as tests of normality and adjustment for multiple comparisons
☐	☒	A full description of the statistical parameters including central tendency (e.g. means) or other basic estimates (e.g. regression coefficient) AND variation (e.g. standard deviation) or associated estimates of uncertainty (e.g. confidence intervals)
☐	☒	For null hypothesis testing, the test statistic (e.g. *F*, *t*, *r*) with confidence intervals, effect sizes, degrees of freedom and *P* value noted<br>*Give P values as exact values whenever suitable.*
☒	☐	For Bayesian analysis, information on the choice of priors and Markov chain Monte Carlo settings
☒	☐	For hierarchical and complex designs, identification of the appropriate level for tests and full reporting of outcomes
☒	☐	Estimates of effect sizes (e.g. Cohen's *d*, Pearson's *r*), indicating how they were calculated

Our web collection on statistics for biologists contains articles on many of the points above.

Software and code

Policy information about availability of computer code

Data collection

For single cell RNA sequencing assay, cells from unfractionated tumor were encapsulated using 10X Genomics' Chromium controller and Single Cell 3' Reagent Kits v2. Following capture and lysis, cDNA was synthesized and amplified to construct Illumina sequencing libraries. The libraries from about 1,000 cells per sample were sequenced with Illumina Nextseq 500.

Data analysis

Single cell RNA sequencing data were processed by the Sequencing and Microarray Facility in MD Anderson Cancer Center. Library Seurat (version 3.5.3), dplyr (version 1.0.7) and cowplot (version 1.1.1) were loaded into R (version 3.6.1) to explore QC metrics, filter cells, normalize data, cluster cells, and identify cluster biomarkers. Data statistical analyses were performed using GraphPad Prism (version 8.0.0). The analysis of microCT and X-ray imaging was conducted using SkyScan DataViewer software version 1.5.0 (Bruker). The biomechanics analysis was conducted using Bluehill Software version 2.9 (Instron). Confocal microscopic images were analyzed with ZEN software version 2.1 (Zeiss).

For manuscripts utilizing custom algorithms or software that are central to the research but not yet described in published literature, software must be made available to editors and reviewers. We strongly encourage code deposition in a community repository (e.g. GitHub). See the Nature Portfolio guidelines for submitting code & software for further information.

Data

Policy information about availability of data

All manuscripts must include a data availability statement. This statement should provide the following information, where applicable:

- Accession codes, unique identifiers, or web links for publicly available datasets
- A description of any restrictions on data availability
- For clinical datasets or third party data, please ensure that the statement adheres to our policy

The authors declare that all data supporting the findings of this study are available within the paper and the supplementary information. Single-cell RNA-sequencing

source data generated by this study can be accessed with the accession number GEO: GSE184360. Single-cell RNA-sequencing source data of a previously published dataset can be accessed with the accession number GEO: GSE128423.

Field-specific reporting

Please select the one below that is the best fit for your research. If you are not sure, read the appropriate sections before making your selection.

☒ Life sciences ☐ Behavioural & social sciences ☐ Ecological, evolutionary & environmental sciences

For a reference copy of the document with all sections, see nature.com/documents/nr-reporting-summary-flat.pdf

Life sciences study design

All studies must disclose on these points even when the disclosure is negative.

Sample size	The sample size of the experiments was based on our previous experiments with transgenic mouse models including the Col1a1loxp/loxp strain (Chen et al., Cancer Cell, 2021, PMID: 33667385). The sample size for each experiment is mentioned in the figure legends. Sample size of the number of independently interrogated biological samples was limited by the number of obtained transgenic mice with the desired genotype. The sample size for the X-ray imaging, microCT imaging, biomechanics test, and immunostaining experiments was at least three biological replicates (as biologically independent samples from individual mice) per group.
Data exclusions	No samples/animals was excluded from any analysis.
Replication	The experiments using mouse models were confirmed with multiple technical and biological replicates, as stated in the methods. All attempts at replication were successful, and the phenotypes of indicated transgenic mouse models with Col1a1 knockout were consistently observed through the 4-year time span of our study. The frequency of examination on independent biological replicates was based on the breeding of transgenic mice to generate the experimental mice with desired genotypes. The bone phenotypes of experimental mice (with each mouse as an independent biological replicate) are examined by X-ray on a monthly basis after the mice reach weaning age. Different batches of experimental mice (born at different time) were examined independently but using the same parameter settings.
Randomization	No randomization was used for animal studies. In this study, we compared the bone phenotypes of transgenic mice with Col1a1 genetic deletion to wild-type control littermates. These experimental mice (with conditional knockout) and wild-type control mice were born with their group/genotype identities, making the randomization impossible and unnecessary.
Blinding	Investigators were not blinded for group identification or genotype identification for the transgenic mouse models used in this study. As part of the standard protocols for transgenic mouse maintenance, the group identification and genotype identification of experimental transgenic mice and wild-type control littermates must be identified by genotyping PCRs at the age of 3 weeks, therefore making the blinded of groups impossible. However, the terminal operator was blinded at the time of body weight measurement, X-ray imaging, micro-CT imaging, biomechanical examinations, and other assays.

Behavioural & social sciences study design

All studies must disclose on these points even when the disclosure is negative.

Study description	NA
Research sample	NA
Sampling strategy	NA
Data collection	NA
Timing	NA
Data exclusions	NA
Non-participation	NA
Randomization	NA

Ecological, evolutionary & environmental sciences study design

All studies must disclose on these points even when the disclosure is negative.

Study description	NA

Research sample	NA
Sampling strategy	NA
Data collection	NA
Timing and spatial scale	NA
Data exclusions	NA
Reproducibility	NA
Randomization	NA
Blinding	NA

Did the study involve field work? ☐ Yes ☐ No

Field work, collection and transport

Field conditions	NA
Location	NA
Access & import/export	NA
Disturbance	NA

Reporting for specific materials, systems and methods

We require information from authors about some types of materials, experimental systems and methods used in many studies. Here, indicate whether each material, system or method listed is relevant to your study. If you are not sure if a list item applies to your research, read the appropriate section before selecting a response.

Materials & experimental systems

n/a	Involved in the study	
☐	☒	Antibodies
☒	☐	Eukaryotic cell lines
☒	☐	Palaeontology and archaeology
☐	☒	Animals and other organisms
☒	☐	Human research participants
☒	☐	Clinical data
☒	☐	Dual use research of concern

Methods

n/a	Involved in the study	
☒	☐	ChIP-seq
☒	☐	Flow cytometry
☒	☐	MRI-based neuroimaging

Antibodies

Antibodies used	All antibodies used in this study were commercially available with detailed information provided in the Methods section. The antibodies used in this study are: goat anti-Collagen1 (SouthernBiotech, 1310-01, lot#B2918-XB89, 1:200), biotinylated donkey anti-goat secondary antibody (Jackson ImmunoResearch, 705-066-147, lot#127411, 1:400), rabbit anti-GFP/YFP (Abcam, ab290, lot#GR3321614-1, 1:500), chicken anti-GFP/YFP (Abcam, ab13970, lot# GR3190550-13, 1:400), rabbit anti-osteocalcin/OCN (Millipore/Sigma-Aldrich, ab10911, lot#3670782, 1:200), rat anti-Ly6G (Abcam, ab25377, clone RB6-8C5, lot#GR3391587-2, 1:100), mouse anti-RFP/tdTomato (Thermo Fisher, MA5-15257, clone RF5R, lot#SD247965, 1:100), and AF488/594-labeled secondary antibodies: Thermo Fisher A11012 (lot#1933366), A11039 (lot#2079383), A11008 (lot#1937184), and A21203 (lot#1722995), all in 1:400 dilution.
Validation	All antibodies used in this study were previously confirmed by the manufacturers and previous research studies that can be found on the manufacturers' websites. Goat anti-type I collagen antibody was validated for the application of immunohistochemistry staining on paraffin sections (with mouse reactivity). Rabbit and chicken anti-GFP/YFP antibodies were validated for the application of immunohistochemistry staining on paraffin sections. Rabbit anti-osteocalcin antibody was validated for the application of immunohistochemistry staining (with mouse reactivity). Rat anti-Ly6G antibody was validated for the application of immunohistochemistry staining (with mouse reactivity).

Eukaryotic cell lines

Policy information about cell lines

Cell line source(s)	NA
Authentication	NA
Mycoplasma contamination	NA
Commonly misidentified lines (See ICLAC register)	NA

Palaeontology and Archaeology

Specimen provenance	NA
Specimen deposition	NA
Dating methods	NA

☐ Tick this box to confirm that the raw and calibrated dates are available in the paper or in Supplementary Information.

Ethics oversight	NA

Note that full information on the approval of the study protocol must also be provided in the manuscript.

Animals and other organisms

Policy information about studies involving animals; ARRIVE guidelines recommended for reporting animal research

Laboratory animals	All mice were housed under standard housing conditions at MDACC animal facilities, and all animal procedures were reviewed and approved by the MDACC Institutional Animal Care and Use Committee. Fsp1-Cre and αSMA-Cre mouse strains were previously documented (Chen et al., EMBO Mol Med. 2018. PMID: 30120146). Cdh5-Cre (017968; B6.129-Tg-Cdh5-cre-1Spe/J), CMV-Cre (006054; B6.C-Tg-CMV-cre-1Cgn/J), and LSL-YFP (006148; B6.129X1-Gt-ROSA-26Sortm1-EYFP-Cos/J) mouse strains were purchased from Jackson Laboratory. Osteogenesis imperfecta murine (OIM) strain harboring Col1a2 mutation (001815; B6C3Fe a/a-Col1a2oim/J) was purchased from Jackson Laboratory. Col1a1loxP/loxP mouse strain (with loxP-flanked exons 2-5) was established in the Genetically Engineered Mouse Facility at MD Anderson Cancer Center (MDACC) using the Col1a1 tm1a (EUCOMM) Wtsi embryonic stem cells that were obtained from the European Mouse Mutant Cell Repository (EuMMCR), as documented by our previous study (Chen et al., Cancer Cell. 2021. PMID: 33667385). Both female and male mice with desired genotype(s) were used for experimental mice.
Wild animals	No wild animals were used in the study.
Field-collected samples	No field-collected samples were used in the study.
Ethics oversight	The experiments were reviewed and approved by IACUC of MDACC.

Note that full information on the approval of the study protocol must also be provided in the manuscript.

Human research participants

Policy information about studies involving human research participants

Population characteristics	NA
Recruitment	NA
Ethics oversight	NA

Note that full information on the approval of the study protocol must also be provided in the manuscript.

Clinical data

Policy information about clinical studies

All manuscripts should comply with the ICMJEguidelines for publication of clinical research and a completedCONSORT checklist must be included with all submissions.

Clinical trial registration	NA
Study protocol	NA

Data collection	NA
Outcomes	NA

Dual use research of concern

Policy information about dual use research of concern

Hazards

Could the accidental, deliberate or reckless misuse of agents or technologies generated in the work, or the application of information presented in the manuscript, pose a threat to:

No	Yes	
☒	☐	Public health
☒	☐	National security
☒	☐	Crops and/or livestock
☒	☐	Ecosystems
☒	☐	Any other significant area

Experiments of concern

Does the work involve any of these experiments of concern:

No	Yes	
☒	☐	Demonstrate how to render a vaccine ineffective
☒	☐	Confer resistance to therapeutically useful antibiotics or antiviral agents
☒	☐	Enhance the virulence of a pathogen or render a nonpathogen virulent
☒	☐	Increase transmissibility of a pathogen
☒	☐	Alter the host range of a pathogen
☒	☐	Enable evasion of diagnostic/detection modalities
☒	☐	Enable the weaponization of a biological agent or toxin
☒	☐	Any other potentially harmful combination of experiments and agents

ChIP-seq

Data deposition

☐ Confirm that both raw and final processed data have been deposited in a public database such as GEO.

☐ Confirm that you have deposited or provided access to graph files (e.g. BED files) for the called peaks.

Data access links<br>*May remain private before publication.*	NA
Files in database submission	NA
Genome browser session<br>(e.g. UCSC)	NA

Methodology

Replicates	NA
Sequencing depth	NA
Antibodies	NA
Peak calling parameters	NA
Data quality	NA
Software	NA

Flow Cytometry

Plots

Confirm that:

- [ ] The axis labels state the marker and fluorochrome used (e.g. CD4-FITC).
- [ ] The axis scales are clearly visible. Include numbers along axes only for bottom left plot of group (a 'group' is an analysis of identical markers).
- [ ] All plots are contour plots with outliers or pseudocolor plots.
- [ ] A numerical value for number of cells or percentage (with statistics) is provided.

Methodology

Sample preparation	NA
Instrument	NA
Software	NA
Cell population abundance	NA
Gating strategy	NA

- [ ] Tick this box to confirm that a figure exemplifying the gating strategy is provided in the Supplementary Information.

Magnetic resonance imaging

Experimental design

Design type	NA
Design specifications	NA
Behavioral performance measures	NA

Acquisition

Imaging type(s)	NA
Field strength	NA
Sequence & imaging parameters	NA
Area of acquisition	NA

Diffusion MRI ☐ Used ☐ Not used

Preprocessing

Preprocessing software	NA
Normalization	NA
Normalization template	NA
Noise and artifact removal	NA
Volume censoring	NA

Statistical modeling & inference

Model type and settings	NA
Effect(s) tested	NA

Specify type of analysis: ☐ Whole brain ☐ ROI-based ☐ Both

Statistic type for inference (See Eklund et al. 2016)	NA
Correction	NA

Models & analysis

n/a | Involved in the study

- [ ] [ ] Functional and/or effective connectivity
- [ ] [ ] Graph analysis
- [ ] [ ] Multivariate modeling or predictive analysis

Functional and/or effective connectivity	NA
Graph analysis	NA
Multivariate modeling and predictive analysis	NA
