# Supplementary material for: Type-I collagen produced by distinct fibroblast lineages reveals specific function during embryogenesis and Osteogenesis Imperfecta
Source: Nat Commun. 2021 Dec 10;12:7199. doi: 10.1038/s41467-021-27563-3 (PMC8664945; doi:10.1038/s41467-021-27563-3)
Supplement: Supplementary file 3 — Description of Additional Supplementary Files [file 41467_2021_27563_MOESM3_ESM.pdf]

Title: Supplementary Movie 1. Movement of 6-month-old $Col1a1^{fspKO}$ and WT mice.

Description: Representative video showing the impaired movement due to spontaneous Osteogenesis Imperfecta phenotype in a 6-month-old $Col1a1^{fspKO}$ mouse (located in the upper right corner at the beginning of the video), as compared with age-matched WT littermate mouse (located in the lower left corner at the beginning of the video).
